# Global burden and trends of pelvic organ prolapse associated with aging women: An observational trend study from 1990 to 2019

**DOI:** 10.3389/fpubh.2022.975829

**Published:** 2022-09-15

**Authors:** Bo Wang, Yingying Chen, Xiaoran Zhu, Tian Wang, Mei Li, Yibao Huang, Liru Xue, Qingqing Zhu, Xiaofan Gao, Mingfu Wu

**Affiliations:** Key Laboratory of Cancer Invasion and Metastasis, Ministry of Education, Department of Gynecology, National Clinical Research Center for Obstetrical and Gynecological Diseases, Tongji Hospital, Tongji Medical College, Huazhong University of Science and Technology, Wuhan, China

**Keywords:** aging, pelvic organ prolapse, epidemiology, incidence, disability adjusted life years

## Abstract

**Purpose:**

Worldwide, about 40% of women will experience pelvic organ prolapse (POP), and this proportion is expected to increase with the aging of the population. We investigated the global, regional and national influenza burden in the past 30 years through the age and sociodemographic index (SDI).

**Patients and methods:**

Data were extracted from the Global Burden of Disease (GBD) 2019 database for 195 countries and territories between 1990 and 2019. Estimated annual percentage changes (EAPCs) were used to explore the age-standardized incidence rate (ASIR) and age-standardized disability adjusted life years (AS-DALYs) trends, and the corresponding 95% uncertainty intervals (UI). In addition, the time cut-off points of 1990 and 2019 were used to separately analyze the incidence rate and DALYs.

**Results:**

In 2019, the global ASIR and AS-DALYs for POP were 316.19 (95%UI: 259.84–381.84) and 10.37 (95%UI: 5.79–17.99) per 100,000 population, respectively. Moreover, from 1990 to 2019, the ASR of both showed a downward trend, and EAPCs were −0.46 (95%CI: −0.52 to −0.4) and −0.53 (95%CI: −0.58 to −0.47), respectively. In addition, DALYs of POP also showed a downward trend in most regions and countries with high SDI. From 1990 to 2019, the global incidence rate and DALYs rate were highest in the 65–75 and ≥60 age groups, respectively.

**Conclusion:**

Over the past three decades, the incidence and DALY of POP have been decreasing from 1990 to 2019. However, POP remains a major health problem, especially among females in less developed countries. Primary and secondary prevention measures of POP should be integrated into the practice of healthcare professionals dealing with aging women.

## Introduction

The aging process has always been the main risk factor for the development of age-related diseases (1, 2). The World Health Organization (who) has issued disease standards to apply to the aging assessment of human organisms, which directly defines aging phenomena and can be classified as diseases (3–5).Worldwide, pelvic organ prolapse (POP) is defined as descent of pelvic organs from the normal anatomic position usually to or beyond the hymenal remnants, owing to loss of support from the connective tissue, muscles, or both. It can lead to symptoms of pelvic pressure, vaginal bulge, urinary and bowel dysfunction, and sexual dysfunction in elderly patients (6–8). However, aging was the most frequently reported risk factor for POP, followed by parity and obesity (9). It is reported that the proportion of women aged 70–79 seeking medical consultation due to symptomatic POP is the highest, as high as 18.6/1,000 (10, 11). And most remarkably, POP remains a problem even in high-income countries, as shown in the United States, the annual incidence rate of POP is 1.5–1.8/1,000, and the highest incidence rate is among women aged 60–69 (11, 12). Given the aging population in the United States, the number of women suffering from POP is expected to increase by about 50% by 2050 (6). A study from the Gambia found that 46% of women had some degree of prolapse on examination, but only 12.5% of the women reported symptoms related to POP (13). Another study predicted that the total number of women who will undergo surgery for POP disorders will increase 48.1% by 2050 due to the aging population (14). Undoubtedly, the development of POP disrupts the quality of life (QoL) and damages social and personal activities. In general, the QoL among women with prolapse was worse than that of the age-standardized population. Although most patients perceived their condition to be improved after non-surgical and surgical treatment (15), POP is still highly prevalent among rural women and remains untreated (16). In general, POP, as the most important factor affecting the health quality of elderly women, its incidence rate has attracted more and more attention, and taking necessary prevention and control strategies is the fundamental to truly improve the health quality of these women.

To date, the treatment of POP depends on the symptoms, type, and grade of prolapse and any related medical complications, including observation, non-surgical and surgical techniques (9). Management should be individualized and guided by what the patient wishes to achieve. Notably, surgical treatments are often performed in the women with more severe prolapse and associated symptoms. For example, transvaginal bilateral sacrospinous fixation appears to be safe and effective to improve both the QoL and sexual functions in patients with second recurrence of vaginal vault prolapse (17, 18). Da-Vinci robotic system was also feasible for the treatment of pelvic organ prolapse with 95% surgical success rate (19). In addition, biocompatible porcine dermis graft to treat severe cystocele considerably improves the QoL and sexual function, and does not influence clitoral blood flow (20). However, the management of POP worldwide is a common and challenging task, especially since the exact prevalence is difficult to establish. In addition, the severity and degree of symptoms can vary widely, so it is difficult to determine and define “treatment success.” To some extent, the diagnosis and treatment of pop still need to be continuously optimized and improved. Up to now, there is still a long way to go.

According to existing studies, the reported prevalence of POP varies widely, ranging from 3 to 50% (21–23). Therefore, up-to-date and comprehensive evidence in this regard is essential for the development of intervention strategies, especially epidemiological investigations are urgently needed on a variety of issues, including women's views on prolapse symptoms and the exact incidence trend. Fortunately, the global burden of disease (GBD) studies have derived detailed and comparable epidemiological and burden of disease estimates for POP (24).

As with most non-communicable diseases, a better understanding of the epidemiology of pelvic floor dysfunction may help to improve the effectiveness of prevention and treatment. Given this, the study introduces the global burden and trends of GBD 2019 research, describes the incidence rate, life lost year (YLS), disability year (YLDs), and disability-adjusted life year (DALYs) in 195 countries (including the time nodes in 1990 and 2019, and the trend of EAPC over 30 years), and provides information for POP control through policy, resource allocation and health system planning.

## Materials and methods

### A framework of the 2019 GBD study

**Figure 1:**
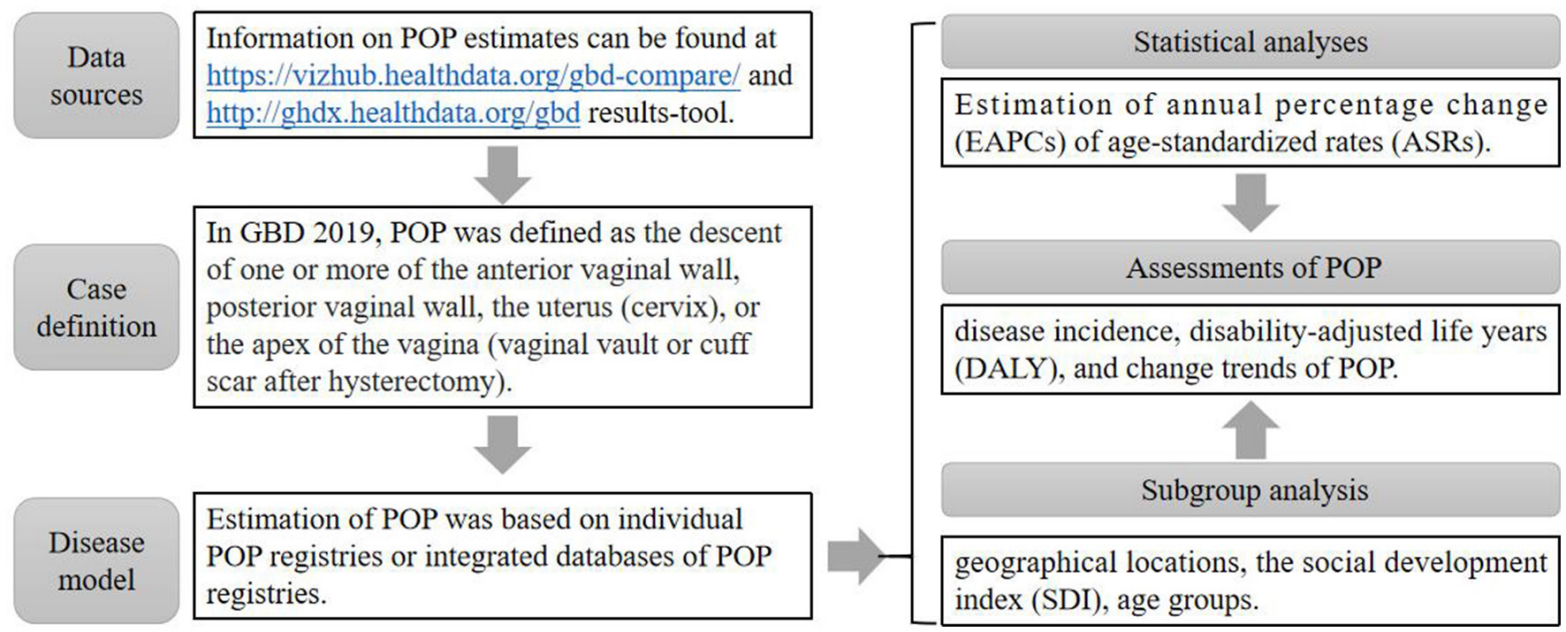
Flow chart of GBD data acquisition. Notes. ASR is assumed to be linear along with time; that is, *Y* = α + β*X* + ε, where Y refers to ln (ASR), X the calendar year, and ε the error term. Based on this formula, β represents the positive or negative ASR trends. The EAPC was calculated as EAPC = 100 × (exp(β)-1). Its 95% confidence intervals (CI) could be obtained from the linear model. Disability-adjusted life-years for any corresponding subpopulation of a specific cause was the sum of the corresponding YLDs and YLLs. YLLs: years of life lost; YLDs: years lived with disability. Social development index (SDI). The SDI, which is based on national-level income per capita, average years of education among persons older than 15, and total fertility rate, was used to categorize the countries into five SDI quintiles (high, high-medium, medium, low-medium, and low levels).

In this study, we obtained the data from GBD 2019 using the online Global Health Data Exchange (GHDx) query tool (http://ghdx.healthdata.org/gbd-results-tool), including incidence cases, DALY across 195 countries (e.g., taking 1990 and 2019 as time nodes, the 2 years were analyzed separately), as shown in Figure 1. To further investigate the global burden of POP, the social-demographic index (SDI) was used to classify these countries and regions into five categories, namely high SDI, high-middle SDI, middle SDI, and low-middle SDI, and low SDI (25, 26). Additionally, we also drew the world map to observe the incidence rate and DALY of POP in 195 countries, and the corresponding trend in different countries and regions over the three decades. This study complies with the provisions of the Helsinki Declaration (revised in 2013) and was approved by the Institutional Review Committee of Tongji Hospital, Tongji Medical College, Huazhong University of Science and Technology (TJ-IRB20210631).

### Statistical analysis

To evaluate trends of incidence and burden for POP, we calculated relevant evaluation indicators, namely the annual age-standardized incidence rate (ASIR) and age-standardized DALY rate (AS-DALY), and the corresponding estimated annual percentage changes (EAPCs). Among them, DALY is the sum of years lived with disability (YLD) and years of life lost (YLLs) (27). EAPC is a widely used index that describes the trend of ASR (28), which can be calculated using a linear regression model as follows:


$$\begin{array}{l} \;ln\;\left(ASR\right)\;=\;\alpha +\beta \;x+\;\varepsilon ,\\ EAPC\;=\;100\;\times \;\left(exp\;\left(\beta \right)\;-\;1\right). \end{array}$$

In where x refers to the calendar year, and the ASR was obtained as follows:


$$\begin{array}{l} ASR=\frac{\sum_{i=1}^{A}aiwi}{\sum_{i=1}^{A}wi}{\;}^{*}100,000.\; \end{array}$$

(In the *i*th age subgroup, *a*_*i*_is represented as age class. *w*_*i*_ denotes the number of persons (or weight), where *i* is equal to the selected reference standard population) (29). Meanwhile, the judgments of trends were the follows: (1) an increasing trend of ASR was found when both the EAPC value and its 95% CI >0; (2) a decreasing trend of ASR was found when both the EAPC value and 95% CI <0; and (3) any other trends meant the ASR was stable over time (30, 31). In general, EAPC can be used to evaluate the ASIR and AS-DALY of POP over the past 30 years. At the same time, a cross-sectional comparison of the incidence rate and DALY before and after 30 years was made at the time points of 1990 and 2019.

In addition, the human development index SDI is a sociodemographic variable to help differentiate countries to classify the world population in homogeneous groups through more comprehensive indicators (25), which can be used as the evaluation index of health care level in each country. We also used a scatter diagram for visualization to depict the correlation between EAPC, ASR, and SDI, in which the Pearson's correlation coefficient (*R*) represents the strength of the correlation. All analysis was performed using the Python programming language (version 3.9.2, Python Software Foundation, https://www.python.org/) and R Project for Statistical Computing (version 4.0.4, http://www.r-project.org/).

## Results

### Age-standardized incidence rate trends of POP

At the global level, there were 13 million (95% UI: 11–16) incident cases of POP in 2019, with an age-standardized incidence rate of 316.19 per 100,000 population (95% UI: 259.84–381.84), which is 0.85% lower than in 1990 (95% UI: 304.93–455.87).

**Figure 2:**
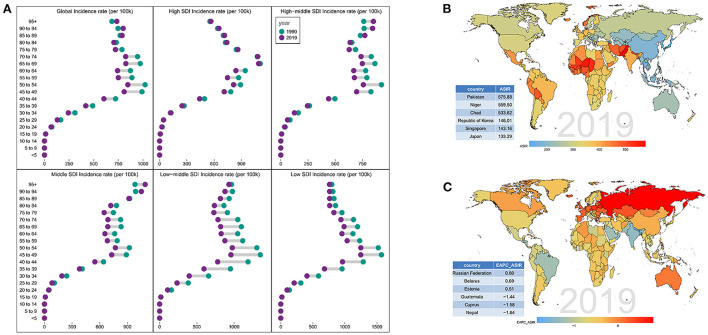
Distribution of incidence rate in different SDI regions. **(A)** Age distribution of incidence rate in different SDI regions from 1990 to 2019. Distribution of **(B)** ASIR and **(C)** EAPC-ASIR in various countries and regions in 2019.

As for age distribution, the ratio of female incidence among different ages showed a bimodal distribution, with peaks in the 50–54 years and 65–69 years age groups (Figure 2). Regarding SDI level analysis, the ASIR in the low SDI region was on the decline with EAPCs of –0.79 (95% CI, from –0.87 to –0.72). The ASIR in the other four SDI regions was stable. In addition, we found that the higher the SDI, the lower the proportion of elderly incidence cases among all POP incidence cases, while the proportion of young incident cases was relatively stable. The proportion of annual young incidence cases decreased year by year, while the proportion of elderly incident cases increased year by year, as shown in Table 1 and Supplementary Figure 1.

**Table 1:** Trends of incidence and age-standardized incidence rate of POP from 1990 to 2019.

	**1990**		**2019**		**1990–2019**
	Incidence cases No. *10^2^ (95% UI)	**ASIR per 100,000 No. (95% UI)**	Incidence cases No. *10^2^ (95% UI)	**ASIR per 100,000 No. (95% UI)**	**EAPC No. (95% CI)**
Overall	84,122.61 (68,784.94–101,956.8)	374.13 (304.93–455.87)	134,356.17 (110,470.39–162,580.97)	316.19 (259.84–381.84)	−0.46 (−0.52 to −0.4)
**Socio-demographic index**				
High SDI	16,005.37 (12,733.72–19,681.84)	309.2 (247.98–379.37)	22,790.44 (18,346.29–27,452.41)	286.75 (234.02–345.28)	−0.24 (−0.29 to −0.19)
High-middle SDI	17,239.01 (14,062.61–20,954.33)	294.9 (241.4–356.99)	25,882.63 (21,158.29–31,201.37)	254.6 (208.21–307.24)	−0.2 (−0.38 to −0.02)
Middle SDI	20,399 (16,780.84–24,700.03)	327.85 (269.43–397.11)	38,191.7 (31,304.14–46,301.37)	279.64 (230.45–338.18)	−0.41 (−0.47 to −0.34)
Low-middle SDI	21,267.98 (17,224.96–25,625.5)	544.75 (443.96–663.8)	31,151.71 (25,371.07–37,953.98)	384.71 (313.01–466.05)	−1.13 (−1.16 to −1.09)
Low SDI	9,167.83 (7,377.82–11,170.26)	573.76 (462.15–704.91)	16,266.27 (13,015.84–20,002.25)	450.56 (363.13–552.97)	−0.79 (−0.87 to −0.72)
**Region**				
Andean Latin America	752.35 (593.55–935.44)	570.95 (451.81–713.18)	1,406.77 (1,103.42–1,782.06)	451.87 (354.36–572.57)	−0.76 (−0.83 to −0.69)
Australasia	318.23 (244.98–401.76)	274.24 (211.24–348.74)	558.07 (430.09–707.5)	257.9 (198.76–328.94)	−0.01 (−0.16 to 0.14)
Caribbean	691.35 (539.28–868.42)	463.09 (359.65–581.46)	998.7 (782.05–1,267.13)	380.78 (297.54–483.43)	−0.59 (−0.64 to −0.54)
Central Asia	631.8 (476.27–825.04)	236.72 (179.98–308.55)	974.58 (731.89–1,290.72)	209.28 (159.45–272.78)	−0.03 (−0.31 to 0.25)
Central Europe	2,216.68 (1,700.76–2,828.08)	291.54 (226.04–369.79)	2,681.38 (2,078.41–3,339.39)	285.18 (223.88–357.91)	0.2 (−0.15 to 0.55)
Central Latin America	2,491.91 (2,005.08–3,087.69)	481.39 (384.53–595.59)	5,262.08 (4,223.73–6,493.41)	395.48 (317.52–484.57)	−0.87 (−0.97 to −0.78)
Central Sub-Saharan Africa	680.35 (522.39–871.37)	432.06 (334.42–549.5)	1,443.66 (1,107.3–1,866.74)	373.99 (290.29–484.99)	−0.49 (−0.55 to −0.43)
East Asia	10,962.61 (9,022.15–13,225.68)	229.1 (189.06–275.35)	20,434.6 (16,550.46–24,604.78)	187.39 (153.59–224.11)	−0.29 (−0.45 to −0.12)
Eastern Europe	4,498.95 (3,597.33–5,495.37)	282.68 (230.68–341.88)	4,915.26 (3,890.69–5,941.41)	274.03 (222.36–332.86)	0.63 (0.19 to 1.07)
Eastern Sub-Saharan Africa	2,086.69 (1,650.78–2,590.28)	408.1 (325.88–510.5)	4,257.01 (3,362.62–5,273.88)	354.47 (283.99–439.85)	−0.49 (−0.53 to −0.45)
High-income Asia Pacific	1,755.61 (1,390.22–2,183.16)	156.78 (123.95–194.07)	2,655.29 (2,122.98–3,290.68)	137.27 (109.39–168.4)	−0.46 (−0.58 to −0.35)
High-income North America	5,945.08 (4,766.44–7,117.74)	343.81 (275.78–419.16)	8,939.85 (7,286.23–10,580.24)	308.07 (255.82–364.37)	−0.57 (−0.62 to −0.52)
North Africa and Middle East	5,197.59 (4,016.46–6,647.03)	479.89 (375.84–610.31)	11,315.82 (8,689.89–14,482.75)	404.91 (315.66–517.75)	−0.49 (−0.53 to −0.45)
Oceania	45.19 (34.48–58.56)	251.67 (191.61–323.85)	105.37 (79.69–136.77)	238.8 (180.08–308.62)	−0.19 (−0.2 to −0.18)
South Asia	24,520.07 (19,976.26–29,268.47)	641.36 (524.62–778.36)	35,229.59 (28,670.56–42,515.64)	423.87 (345.17–509.44)	−1.35 (−1.39 to −1.32)
Southeast Asia	3,570.08 (2,877.16–4,422.04)	228.24 (183.13–281.89)	6,923.95 (5,509.33–8,571.86)	193.81 (155–239.76)	−0.54 (−0.58 to −0.5)
Southern Latin America	994.51 (757.57–1,265.74)	403.05 (308.07–517.52)	1,347.59 (1,031.83–1,726.17)	328.04 (251.69–421.67)	−0.6 (−0.67 to −0.54)
Southern Sub-Saharan Africa	669.02 (550.71–812.08)	378.31 (307.77–462.2)	1,191.4 (974.93–1,454.48)	329.6 (269.07–400.74)	−0.48 (−0.49 to −0.47)
Tropical Latin America	3,558.47 (2,886.98–4,258.26)	519.18 (423.03–617.64)	5,079.21 (4,205.11–6,011.8)	389.45 (323.24–459.71)	−1.22 (−1.32 to −1.12)
Western Europe	9,448.94 (7,434.98–11,949.92)	363.72 (284.19–461.37)	11,853.66 (9,357.98–14,826.06)	350.41 (276.38–436.77)	0.05 (−0.06 to 0.16)
Western Sub-Saharan Africa	3,087.15 (2,491.08–3,791.73)	575.92 (464.99–712.95)	6,782.33 (5,455.42–8,406.39)	486.24 (392.93–600.38)	−0.57 (−0.62 to −0.52)

On observation from the GBD regions and countries level, the ASIR showed an upward trend in 27 countries, a stable trend in 145 countries, and a downward trend in 23 countries. Among them, the three countries with the highest ASIR were Russian, Belarus, and Estonia; the three countries with the lowest ASIR were Guatemala, Cyprus, and Nepal (Figure 2 and Supplementary Figure 2). As shown in Supplementary Table 1 and Supplementary Figure 3, most countries had an unimodal age distribution, with peak in elderly (≥65) years age groups.

### Age-standardized DALY rate trends of POP

At the global level, there were 0.27 million (95% UI: 0.15–0.48) DALYs in 1990 and 0.44 million (95% UI: 0.25–0.76) DALYs in 2019. In the past 30 years, the age-standardized DALY rate decreased significantly with an EAPC of −0.53 (95%CI: from −0.58 to −0.47), dropping from 12.39/100,000 persons (95% UI, 6.81–21.45) in 1990 to 10.37/100,000 persons (95% UI, 5.79–17.99) in 2019, as shown in Table 2.

**Table 2:** Trends in disability adjusted life years and age-standardized disability adjusted life years of POP from 1990 to 2019.

	**1990**		**2019**		**1990–2019**
	DALY No. *10^3^ (95% UI)	**Age-standardized DALY Rate per 100,000 No. (95% UI)**	DALY No. *10^3^ (95% UI)	**Age-standardized DALY Rate per 100,000 No. (95% UI)**	**EAPC No. (95% CI)**
Overall	275.96 (154.52–475.31)	12.39 (6.81–21.45)	441.77 (245.44–763.52)	10.37 (5.79–17.99)	−0.53 (−0.58 to −0.47)
**Socio-demographic index**				
High SDI	47.05 (23.53–87.84)	8.58 (4.26–16.01)	67.05 (32.97–125.48)	7.67 (3.75–14.37)	−0.4 (−0.43 to −0.37)
High-middle SDI	49.77 (24.93–92.6)	8.37 (4.17–15.63)	76.74 (38.34–143.43)	7.28 (3.64–13.39)	−0.19 (−0.37 to 0)
Middle SDI	63.6 (35.06–110.72)	10.56 (5.73–18.5)	121.67 (68.03–209.88)	9.04 (5.07–15.77)	−0.42 (−0.49 to −0.36)
Low-middle SDI	82.2 (48.22–136.66)	22.32 (12.99–37.3)	120.19 (70.59–209.16)	15.22 (8.91–26.66)	−1.27 (−1.31 to −1.23)
Low SDI	33.2 (18.74–56.74)	22.7 (12.53–39.13)	55.89 (31.73–94.45)	16.82 (9.49–28.96)	−0.98 (−1.06 to −0.9)
**Region**				
Andean Latin America	2.59 (1.46–4.52)	20.94 (11.36–37.27)	4.55 (2.33–8.32)	14.95 (7.61–27.59)	−1.17 (−1.3 to −1.03)
Australasia	0.94 (0.47–1.72)	7.72 (3.88–14.32)	1.65 (0.78–3.14)	7.03 (3.3–13.44)	−0.17 (−0.34 to 0.01)
Caribbean	2.3 (1.23–4.11)	16.09 (8.6–28.83)	3.37 (1.79–6.21)	12.63 (6.72–23.23)	−0.79 (−0.84 to −0.74)
Central Asia	2.05 (1.12–3.68)	7.29 (3.99–13.14)	2.88 (1.53–5.14)	6.36 (3.43–11.51)	−0.13 (−0.4 to 0.14)
Central Europe	6.7 (3.32–12.47)	8.21 (4.08–15.14)	8.36 (4.04–16.05)	7.85 (3.77–14.91)	0.14 (−0.24 to 0.52)
Central Latin America	7.4 (3.95–13.28)	15.48 (8.18–28.09)	15.21 (7.47–28.57)	11.65 (5.73–21.86)	−1.19 (−1.32 to −1.05)
Central Sub-Saharan Africa	2.28 (1.19–4.03)	16.1 (8.43–28.68)	4.49 (2.44–7.93)	12.95 (6.87–23.1)	−0.71 (−0.81 to −0.6)
East Asia	28.11 (13.7–53.29)	6.06 (2.93–11.48)	55.12 (27.59–102.93)	5.04 (2.56–9.35)	−0.16 (−0.33 to 0.01)
Eastern Europe	13.33 (6.71–25.06)	7.69 (3.89–14.35)	14.72 (7.31–27.86)	7.39 (3.69–13.99)	0.61 (0.17 to 1.04)
Eastern Sub-Saharan Africa	6.07 (3.26–10.74)	13.24 (7.05–23.87)	11.94 (6.16–21.21)	11.16 (5.71–20.32)	−0.59 (−0.63 to −0.55)
High-income Asia Pacific	4.54 (2.3–8.55)	4.04 (2.07–7.52)	7.46 (3.62–13.96)	3.45 (1.7–6.49)	−0.54 (−0.67 to −0.42)
High-income North America	16.28 (8.01–30.55)	8.9 (4.4–16.84)	24.21 (11.85–44.16)	7.65 (3.7–14.1)	−0.84 (−0.96 to −0.72)
North Africa and Middle East	16.01 (8.29–29.27)	15.49 (7.85–28.71)	33.1 (16.3–61.61)	12.87 (6.41–23.92)	−0.52 (−0.56 to −0.48)
Oceania	0.13 (0.07–0.23)	7.57 (3.93–13.74)	0.31 (0.16–0.55)	7.39 (3.89–13.47)	−0.05 (−0.07 to −0.02)
South Asia	101.89 (60.76–170.99)	28.97 (17.03–47.71)	152.39 (91.65–256.86)	18.87 (11.28–31.68)	−1.44 (−1.47 to −1.41)
Southeast Asia	11.07 (6.15–20.02)	7.11 (3.83–12.93)	21.65 (11.94–38.22)	6.19 (3.41–11.02)	−0.47 (−0.49 to −0.44)
Southern Latin America	2.97 (1.46–5.68)	11.85 (5.81–22.64)	4.11 (1.97–7.77)	9.56 (4.58–18.08)	−0.68 (−0.74 to −0.61)
Southern Sub-Saharan Africa	1.91 (0.98–3.52)	11.44 (5.85–21.22)	3.38 (1.72–6.22)	9.76 (4.97–18.02)	−0.54 (−0.56 to −0.52)
Tropical Latin America	10.09 (4.92–18.44)	16.35 (8.06–29.49)	16.14 (8.02–29.74)	12.16 (6.06–22.36)	−1.3 (−1.41 to −1.19)
Western Europe	29.83 (14.9–55.81)	10.47 (5.15–20.08)	38.38 (18.74–72.16)	10.04 (4.81–19.09)	−0.02 (−0.1 to 0.07)
Western Sub-Saharan Africa	9.5 (4.97–17.21)	18.82 (9.55–34.82)	18.35 (9.04–34.08)	14.89 (7.26–28.09)	−0.82 (−0.87 to −0.78)

**Figure 3:**
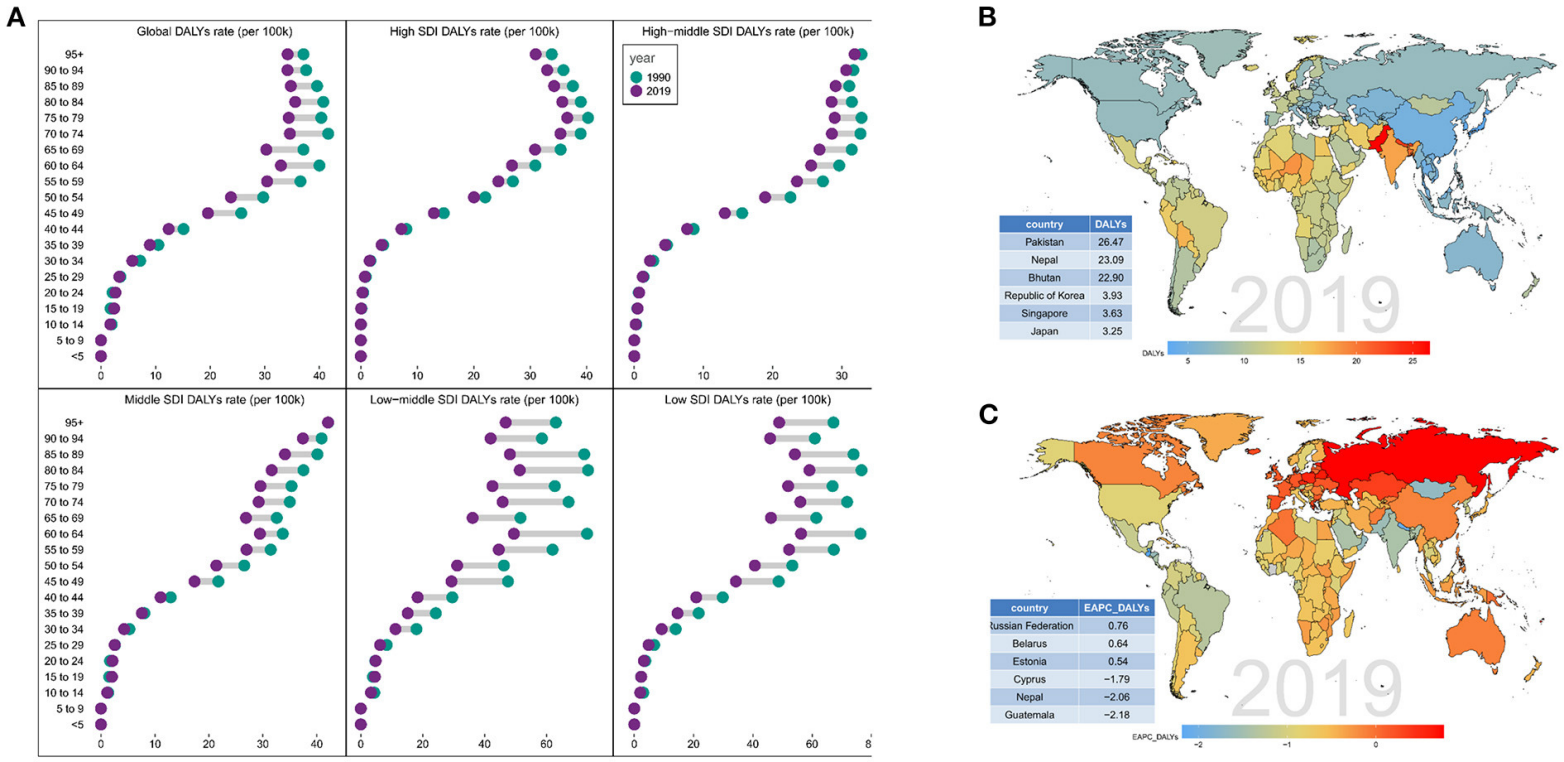
Distribution of DALYs in different SDI regions. **(A)** Distribution of DALYs of different age proportions in different SDI regions from 1990 to 2019. Distribution of **(B)** DALY and **(C)** EAPC-DALY in various countries and regions in 2019.

On analysis from the SDI level, the age-standardized DALY rate in all the SDI regions declined. As for age distribution, the ratio of female incidence among different ages also showed a bimodal distribution, with peaks in 60–64 and 70–74 years age groups (Figure 3 and Supplementary Figure 2). On observation from the GBD regions and countries level, the age-standardized DALY rate in most regions declined and few countries had a rising trend. Among them, the three countries with the highest age-standardized DALY rate were Russian, Belarus, and Estonia; the three countries with the lowest age-standardized DALY rate were Cyprus, Nepal, and Guatemala (Figure 3; Supplementary Table 2; Supplementary Figure 3). Consistent with the trend of ASIR, most countries also showed an unimodal age distribution, with peak in elderly (≥65) years age groups.

### Correlation analysis of POP related ASIR, age-standardized DALYs and different SDI

**Figure 4:**
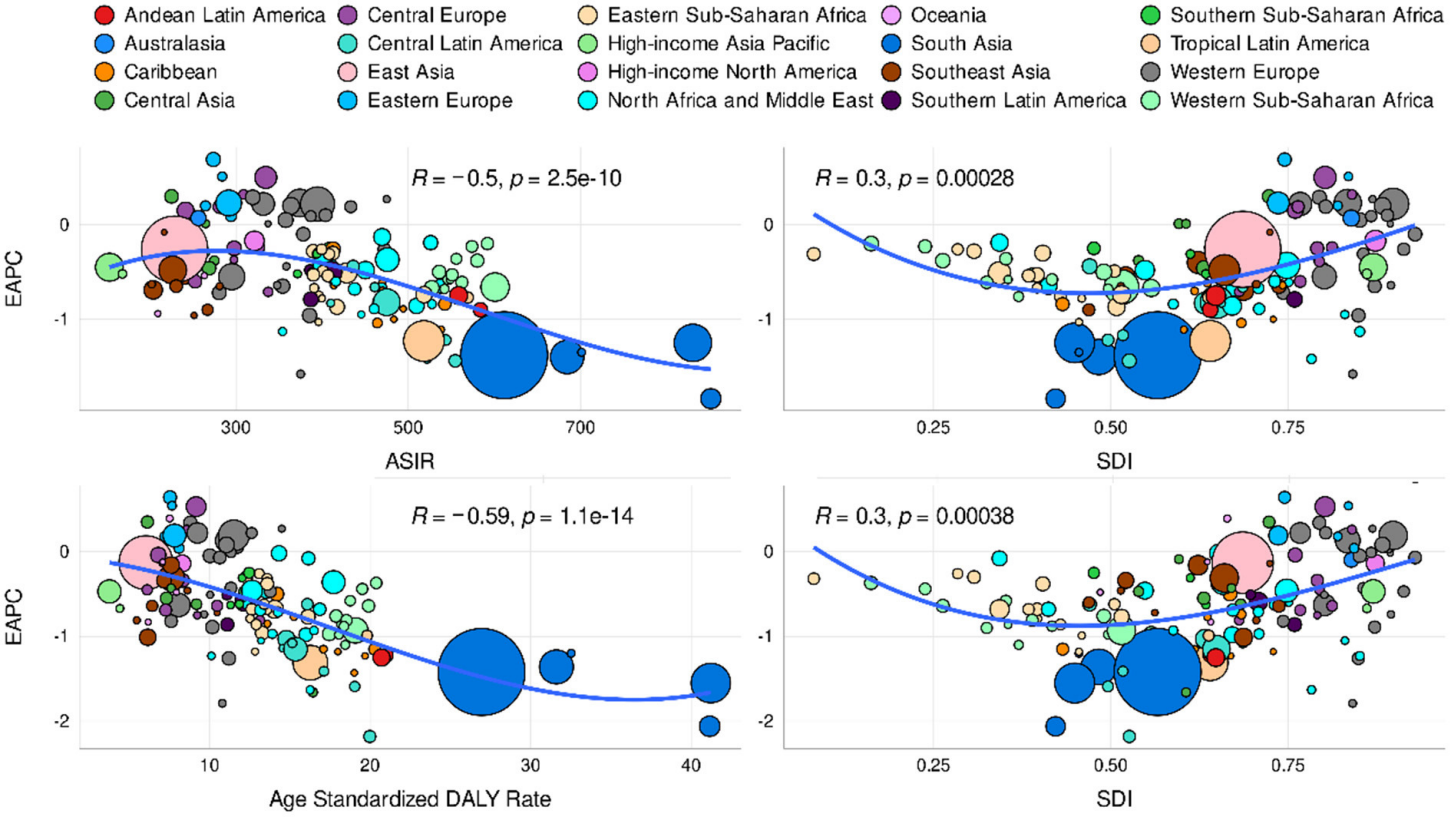
Correlation analysis of different SDI regions, age-standardized DALYs and EAPC of global POP from 1990 to 2019.

Regarding SDI level analysis of ASIR, as shown in Figure 4 and Supplementary Figure 4, we found a positive correlation between EAPC and SDI (*R* = 0.3, *P* < 0.001) and a negative correlation between EAPC and the ASIR (*R* = −0.5, *P* < 0.001). We also found that the higher the SDI, the lower the proportion of young incidence cases among all POP incidence cases, while the proportion of elderly incident cases was relatively stable. In addition, we found a non-significant correlation between EAPC and SDI (*R* = −0.16, *P* = 0.056). As for DALY analysis, we also found a positive correlation between EAPC and SDI (*R* = 0.3, *P* < 0.001) and a negative association between EAPC and the age-standardized DALY rate (*R* = −0.59, *P* < 0.001). The DALY difference among different age groups showed an unimodal distribution, and the peak appeared after 60 years old.

## Discussion

In this study, relying on statistical parameters (ASIR and AS-DALY), we mapped the global epidemic map of POP, which is the first time to obtain authoritative evidence about POP epidemiology based on GBD database. This analysis of the GBD 2019 study shows that there is a considerable POP disease burden globally and in most regions. In general, the incidence rate of age-standardized points and YLD incidence has declined in the past 30 years. Notably, the absolute number of epidemic cases and YLD has increased, which may be mainly due to the population aging and population growth, as well as the improved survival of patients with long-term diseases known to increase the risk of POP. For example, obesity or chronic respiratory diseases increase the intra-abdominal pressure and then contribute to a person's risk of genital prolapse (32). Overall, these trends indicated that POP still poses a huge global health burden, and the overall burden may continue to rise in the future.

Over the past 30 years, due to variations in data sources and methodological differences from the GBD, a comparison to the GBD 1990 and GBD 2019 is not possible. However, it is still feasible to continuously observe the changing trend of diseases. For example, the decrease observed in the age-standardized incidence of POP from 1990 to 2019 was relatively consistent with our findings, where a 0.85% reduction was observed from 1990 to 2019. In addition, the three countries with the highest ASIR were Russian, Belarus, and Estonia; the three countries with the lowest ASIR were Guatemala, Cyprus, and Nepal. Consistent with previous research reports, the POP incidence is much higher in low-income countries, with about 20% previously reported (33, 34). Meanwhile, different studies have shown that in low- and middle-income countries, the average value of pelvic organ prolapse is 19.7%, and the estimated range is 3.4%−56.4% (35). This study gives us great enlightenment, that is, in the environment of uneven global economic development, the uneven distribution of medical and health resources has also had a great impact on POP related chronic diseases. Especially in underdeveloped areas, POP has a high weight in affecting the quality of life of elderly women, while the medical environment in highly developed areas and equipped with advanced diagnosis and treatment system enable elderly women to enjoy more health benefits.

In this study, our results showed that women over the age of 50 bear the largest burden of POP across the world. About 11% of American women have undergone POP or urinary incontinence surgery before the age of 79 and 29.2% of women may need additional surgery (36). In addition, previous literature has reported that POP is very common in women over 40 years, elderly women, and postmenopausal women, with an estimated prevalence of 41%−50% (37, 38). Similar findings also demonstrated that old age, high parity, obesity, vaginal delivery are the most important risk factors leading to POP (35, 39–41). With the increase of age, these factors can complement each other, so aging is an important force of POP risk factors (Supplementary Table 3). For instance, during a woman's first pregnancy, a significant decrease in all compartments of the vaginal wall and perineum was observed, but the total length of the vagina increased with associated pelvic floor dysfunction (42). As documented here, and in previous studies, the global burden of pelvic floor disorder is increasing due to increasing age of the population.

The current research showed that the proportion of annual young incidences in all POP cases decreased year by year, while the proportion of elderly incidences cases increased, which is comparable with previous studies (43, 44). It is well known that aging and fewer births in developed countries have been a serious concern in recent years. Hence, we observed a phenomenon that the higher the SDI, the lower the proportion of young women and the higher the proportion of the elderly, which confirmed this conjecture. For instance, the incident POP population in the high-income Asia Pacific tended to be aging, compared with other regions in Asia. In addition, the improvement of new treatment plans and supportive care measures in developed countries and regions has led to an unprecedented long-term cure rate of POP treatment, which further promotes this trend. In the future health care, the allocation of medical resources should not only focus on young patients with fertility requirements, but also strengthen the monitoring of elderly women (no fertility requirements, but will have a negative impact on the quality of life). Moreover, for postmenopausal women, due to the growth of age and the decline of estrogen levels, these common factors promote the increase of the incidence of POP, so it is more necessary to investigate it as soon as possible.

### Strengths and limitations

Some limitations were unavoidable in this study. First, although GBD includes global pop case data, differences in data collection and coding and data source quality are still inevitable in this analysis model. Second, fluctuations in the incidence rate and DALY annualized rates may partly reflect actual changes in age-related ratios, especially regional differences, which may still be biased. In the future, population-based POP registries must be improved. Third, there are some countries with low socio-economic status and the least data sources, which can also greatly affect the regional burden of estimating POP. Nevertheless, this study presents the latest estimates of the global burden of POP, which will help public health policymakers.

## Conclusion

Over the past three decades, the incidence and DALY of POP have been decreasing from 1990 to 2019. The three countries with the highest ASIR were Russian, Belarus, and Estonia; the three countries with the lowest ASIR were Guatemala, Cyprus, and Nepal. Although the proportion of annual young incidences in all POP cases decreased year by year, the proportion of elderly incidences cases increased. Especially, women over the age of 50 bear the largest burden of POP across the world. Therefore, primary and secondary prevention measures of POP should be integrated into the practice of healthcare professionals dealing with aging women.

## Data availability statement

The original contributions presented in the study are included in the article/Supplementary material, further inquiries can be directed to the corresponding author.

## Ethics statement

Written informed consent was not obtained from the individual(s) for the publication of any potentially identifiable images or data included in this article.

## Author contributions

BW, TW, and MW: study conception and planning, statistical analysis, interpretation of results, manuscript drafting, and final approval of manuscript. XZ, ML, YH, YC, LX, QZ, and XG: interpretation of results and final approval of manuscript. All authors contributed to the article and approved the submitted version.

## Funding

This research was supported by the Applied Basic Research Program of WMBST (no. 2019020701011436).

## Conflict of interest

The authors declare that the research was conducted in the absence of any commercial or financial relationships that could be construed as a potential conflict of interest.

## Publisher's note

All claims expressed in this article are solely those of the authors and do not necessarily represent those of their affiliated organizations, or those of the publisher, the editors and the reviewers. Any product that may be evaluated in this article, or claim that may be made by its manufacturer, is not guaranteed or endorsed by the publisher.

## Supplementary material

Supplementary Figure 1 Incidence rate and DALYs development trend of POP in different SDI regions from 1990 to 2019.

Supplementary Figure 2 Trends of ASIR and age-standardized DALYs in different SDI regions of global POP from 1990 to 2019.

Supplementary Figure 3 EAPC changes of ASIR and age-standardized DALYs in different regions of global POP from 1990 to 2019.

Supplementary Figure 4 Correlation analysis between different SDI regions of global POP and ASIR and DALYs from 1990 to 2019.

Supplementary Table 1 The incidence rate and age standardized incidence rate of POP and the trend of time changes from 1990 to 2019.

Supplementary Table 2 The DALY rate and age standardized DALY rate of POP and the trend of time changes from 1990 to 2019.

Supplementary Table 3 The death and age standardized death rate of POP and the trend of time changes from 1990 to 2019.

Supplementary Table 4 Literature review on risk factors of POP and its recurrence.
