# Supplementary figures and images for: Global burden and trends of pelvic organ prolapse associated with aging women: An observational trend study from 1990 to 2019
Source: Front Public Health. 2022 Sep 15;10:975829. doi: 10.3389/fpubh.2022.975829 (PMC9521163; doi:10.3389/fpubh.2022.975829)

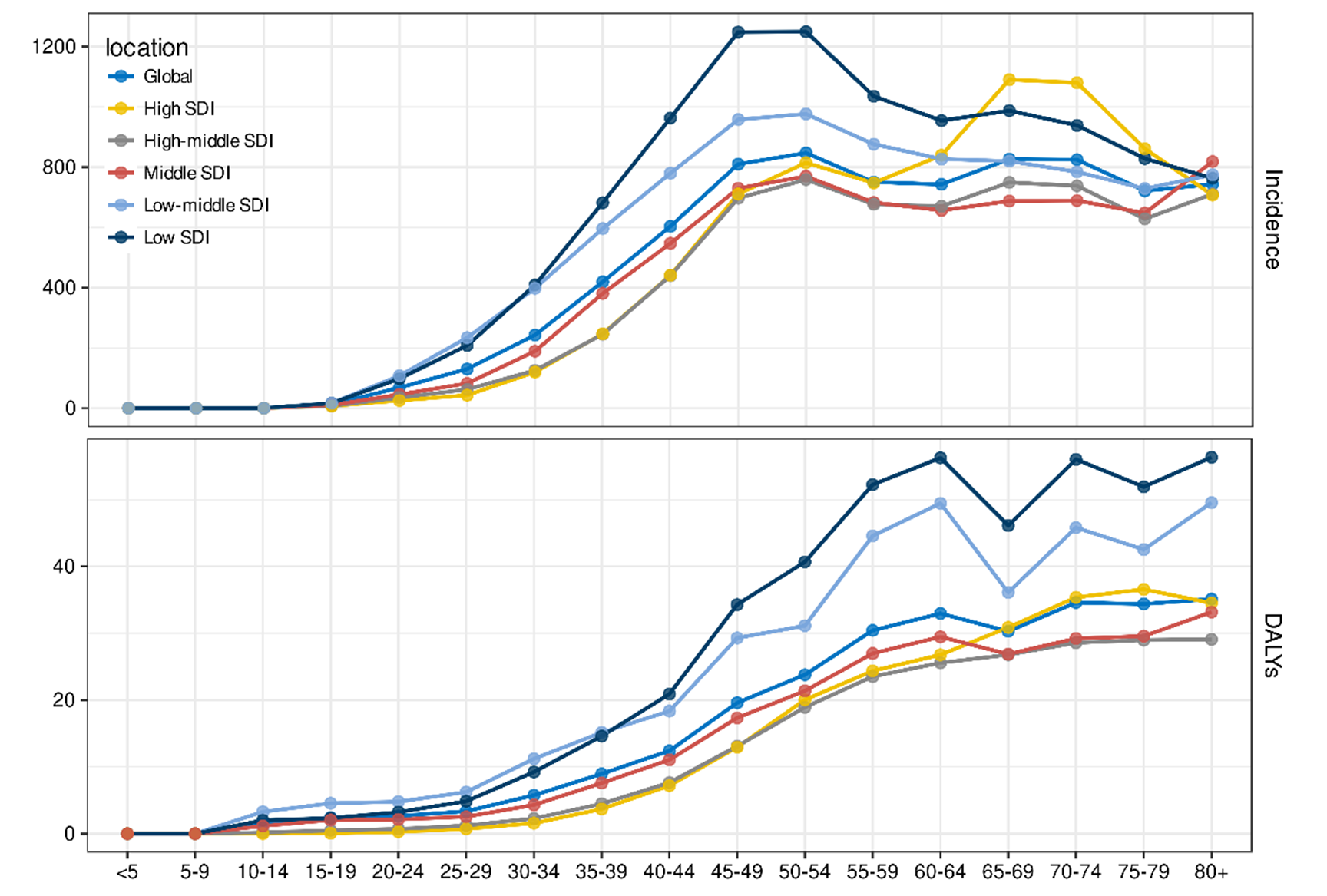

Supplement: Supplementary Figure 1 — Incidence rate and DALYs development trend of POP in different SDI regions from 1990 to 2019. [file Image_1.TIF]

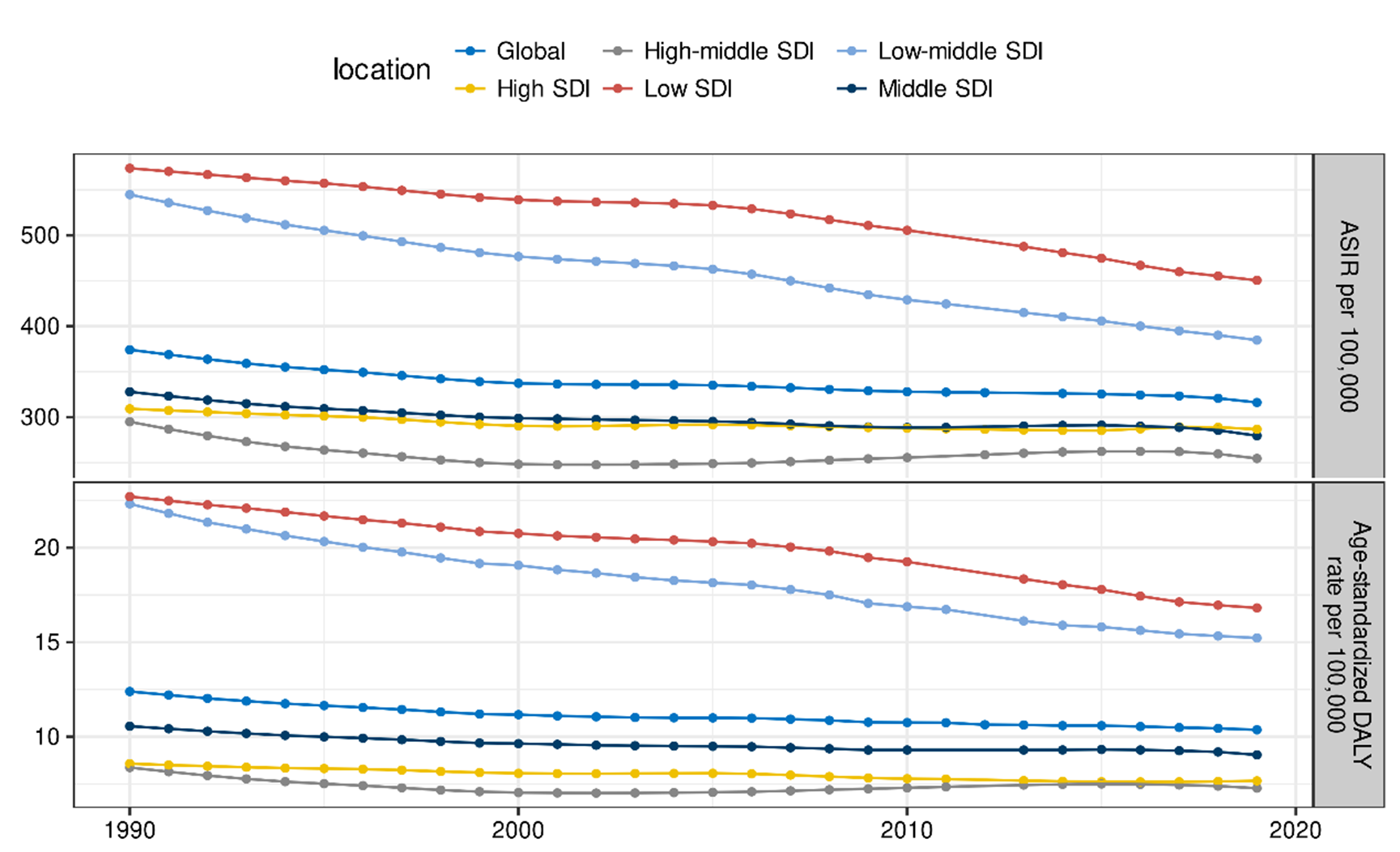

Supplement: Supplementary Figure 2 — Trends of ASIR and age-standardized DALYs in different SDI regions of global POP from 1990 to 2019. [file Image_2.TIF]

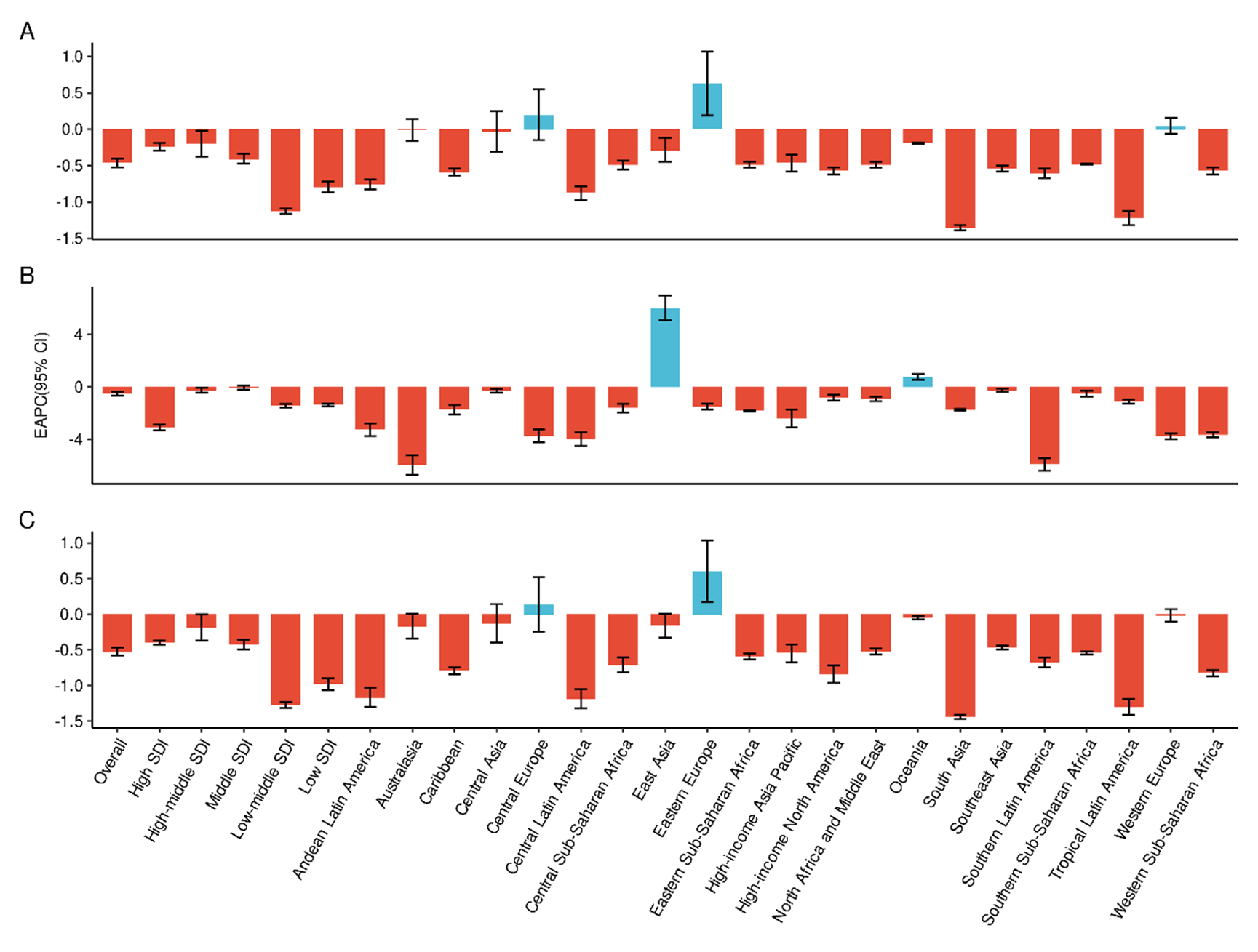

Supplement: Supplementary Figure 3 — EAPC changes of ASIR and age-standardized DALYs in different regions of global POP from 1990 to 2019. [file Image_3.TIF]

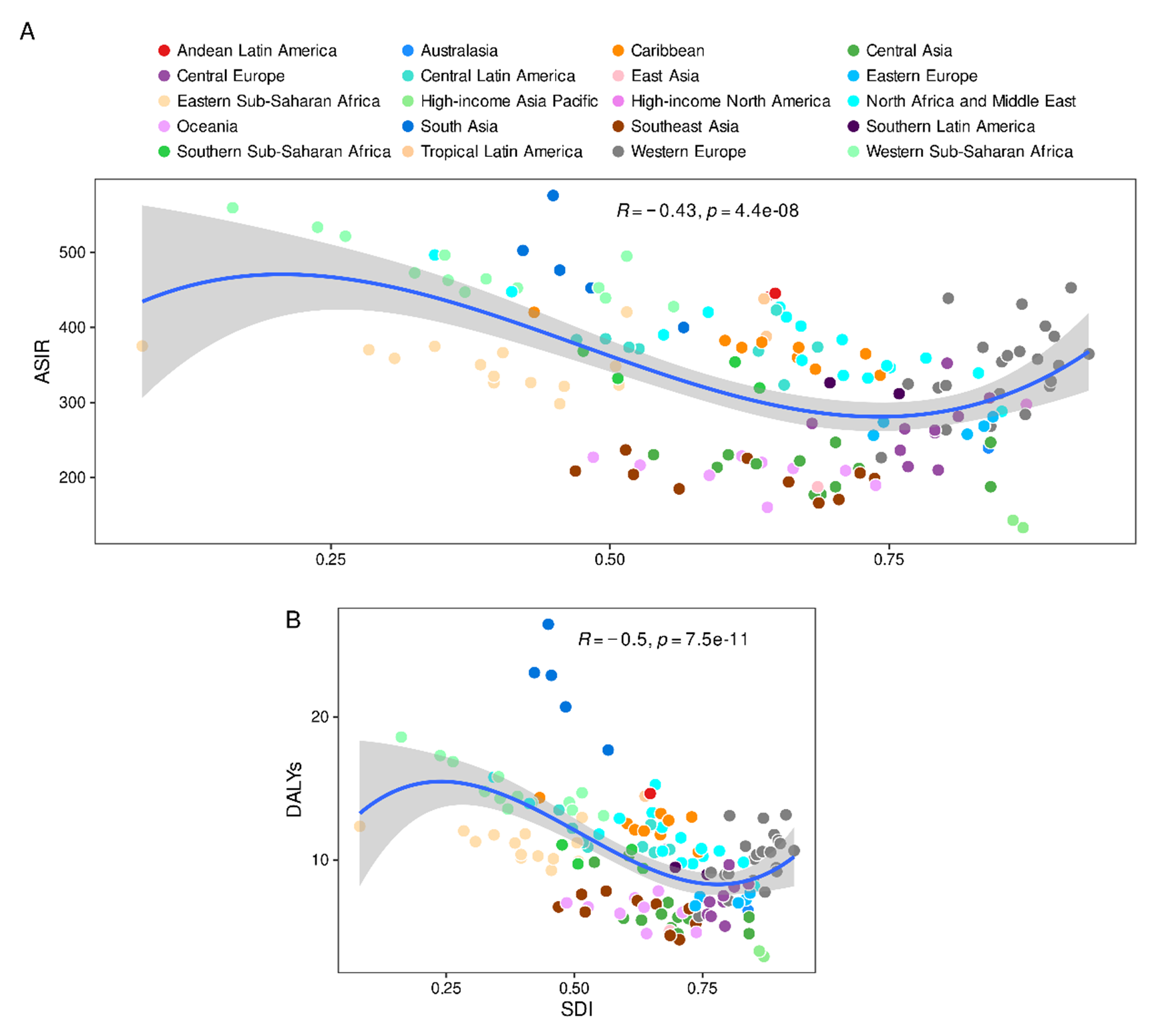

Supplement: Supplementary Figure 4 — Correlation analysis between different SDI regions of global POP and ASIR and DALYs from 1990 to 2019. [file Image_4.TIF]
