# Supplementary material for: Global burden and trends of pelvic organ prolapse associated with aging women: An observational trend study from 1990 to 2019
Source: Front Public Health. 2022 Sep 15;10:975829. doi: 10.3389/fpubh.2022.975829 (PMC9521163; doi:10.3389/fpubh.2022.975829)
Supplement: Supplementary Table 1 — The incidence rate and age standardized incidence rate of POP and the trend of time changes from 1990 to 2019. [file Data_Sheet_1.docx]

Supplementary Table1. The incidence rate and age standardized incidence rate of POP and the trend of time changes from 1990 to 2019

| Nation | Incident cases No.(95% UI) | | Rate of change (%) | ASIR per 100,000 No.(95% UI) | | 1990-2019  EAPC No. (95% CI) |
| --- | --- | --- | --- | --- | --- | --- |
| Afghanistan | 205.24 [156.27-267.86] | 522.35 [389.12-688.1] | 154.51 | 525.98 [403.6-679.25] | 496.66 [383.02-639.81] | -0.19 [-0.23 to -0.15] |
| Albania | 37.75 [28.52-49.74] | 54.27 [40.94-69.71] | 43.76 | 337.29 [254.76-442.25] | 272.18 [204.6-353.02] | -0.71 [-0.88 to -0.54] |
| Algeria | 354.59 [270.11-459.59] | 911.76 [676.48-1169.85] | 157.13 | 469.55 [362.09-603.62] | 427.21 [323.91-546.44] | -0.13 [-0.34 to 0.08] |
| American Samoa | 0.35 [0.27-0.46] | 0.58 [0.43-0.76] | 65.71 | 256.59 [195-330.48] | 216.94 [163.16-279.76] | -0.59 [-0.61 to -0.57] |
| Andorra | 0.96 [0.73-1.24] | 2.04 [1.57-2.66] | 112.50 | 337.14 [257.85-435.53] | 321.7 [248.57-414.01] | 0.01 [-0.08 to 0.11] |
| Angola | 119.48 [90.94-156.26] | 333.5 [254.06-429.3] | 179.13 | 436.03 [336.27-561.64] | 383.85 [296.03-491.08] | -0.44 [-0.48 to -0.41] |
| Antigua and  Barbuda | 1.22 [0.96-1.55] | 1.94 [1.49-2.52] | 59.02 | 433.53 [338.03-553.95] | 347.65 [268.67-447.82] | -0.79 [-0.83 to -0.75] |
| Argentina | 697.4 [528.07-891.69] | 896.23 [686.66-1151.67] | 28.51 | 410.63 [312.1-529.26] | 335.44 [255.38-433.81] | -0.5 [-0.58 to -0.43] |
| Armenia | 33.5 [24.84-43.73] | 38.15 [28.16-50.39] | 13.88 | 216.48 [161.9-283.25] | 177.69 [132.3-234.35] | -0.26 [-0.6 to 0.07] |
| Australia | 248.47 [187.73-318.02] | 436.37 [327.75-563.37] | 75.62 | 256.35 [192.1-330.56] | 239.67 [180.68-309.32] | 0.07 [-0.05 to 0.19] |
| Austria | 206.72 [167.07-245.42] | 225.72 [178.54-276.94] | 9.19 | 385.32 [312.59-456.92] | 311.82 [249.22-385.31] | -0.96 [-1.19 to -0.73] |
| Azerbaijan | 64.89 [48.68-85.71] | 105.67 [78.08-142.7] | 62.84 | 221 [164.77-290.18] | 177.27 [131.76-234.37] | -0.34 [-0.59 to -0.09] |
| Bahamas | 4.78 [3.7-5.99] | 7.77 [5.99-9.98] | 62.55 | 445.36 [347.12-565.64] | 340.88 [264.28-432.54] | -0.98 [-1.04 to -0.93] |
| Bahrain | 5.64 [4.1-7.4] | 23.3 [16.82-30.76] | 313.12 | 415.87 [318.9-544.27] | 346.71 [261.82-447.55] | -0.61 [-0.67 to -0.56] |
| Bangladesh | 2198.39 [1696.14-2766.11] | 3496.94 [2699.42-4406.94] | 59.07 | 684.47 [533.67-857.08] | 452.62 [351.93-571.84] | -1.4 [-1.42 to -1.38] |
| Barbados | 5.71 [4.44-7.15] | 7.23 [5.55-9.2] | 26.62 | 413.54 [321.97-526.83] | 336.38 [257.46-425.61] | -0.64 [-0.74 to -0.54] |
| Belarus | 197.59 [147.56-254.32] | 224.95 [166.97-295.58] | 13.85 | 273.7 [205.22-356.91] | 273.89 [204.49-358.19] | 0.69 [0.32 to 1.06] |
| Belgium | 243.44 [186.21-315.44] | 304.79 [232.89-391.31] | 25.20 | 357.53 [273.34-460.58] | 354.29 [270.91-455.74] | 0.05 [-0.03 to 0.13] |
| Belize | 2.96 [2.31-3.7] | 6.82 [5.27-8.72] | 130.41 | 538.33 [420.84-677.34] | 382.69 [296.74-485.6] | -1.11 [-1.16 to -1.05] |
| Benin | 74.02 [57.89-93.42] | 178.8 [137.62-229.83] | 141.56 | 555.77 [439.88-711.3] | 496.54 [389.83-633.15] | -0.36 [-0.38 to -0.33] |
| Bermuda | 1.43 [1.1-1.82] | 1.7 [1.32-2.17] | 18.88 | 399.79 [307.85-508.93] | 333.76 [258.16-419.92] | -0.52 [-0.64 to -0.4] |
| Bhutan | 13.02 [10.09-16.25] | 16.31 [12.69-20.59] | 25.27 | 701.24 [552.35-868.1] | 476.43 [372.68-603.17] | -1.35 [-1.38 to -1.31] |
| Bolivia  (Plurinational State  of) | 127.87 [98.56-162.2] | 256.83 [199.29-326.03] | 100.85 | 593.61 [464.01-751.82] | 492.07 [384.64-622.65] | -0.58 [-0.65 to -0.5] |
| Bosnia and  Herzegovina | 68 [50.08-89.73] | 66.26 [50.08-86.43] | −2.56 | 278.75 [208.76-368.26] | 237.81 [181.5-312.8] | -0.49 [-0.76 to -0.22] |
| Botswana | 14.27 [10.94-18.24] | 31.71 [24.37-40.87] | 122.21 | 371.38 [288.21-474.95] | 319.49 [247.6-406.61] | -0.52 [-0.53 to -0.5] |
| Brazil | 3471.02 [2815.17-4152.37] | 4931.47 [4101.1-5833.2] | 42.08 | 518.09 [422.89-615.63] | 388.04 [322.13-456.99] | -1.23 [-1.33 to -1.13] |
| Brunei Darussalam | 1.22 [0.92-1.55] | 3.01 [2.21-3.91] | 146.72 | 213.87 [158.52-277.39] | 160.54 [120.56-207.29] | -0.94 [-1.07 to -0.8] |
| Bulgaria | 171.16 [127.34-221.17] | 162.85 [120.25-211.93] | −4.86 | 281.23 [210.69-368.37] | 265.01 [198.17-349.08] | 0.19 [-0.02 to 0.4] |
| Burkina Faso | 164.08 [127.18-211.26] | 356.72 [273.14-456.32] | 117.41 | 567.41 [446.71-727.11] | 515.79 [404.6-655.8] | -0.36 [-0.37 to -0.35] |
| Burundi | 63.8 [48.78-82.05] | 117.68 [89.35-150.87] | 84.45 | 399.45 [309.25-518.97] | 370.37 [285.45-472.6] | -0.26 [-0.28 to -0.24] |
| Cabo Verde | 6.15 [4.84-7.72] | 10.06 [7.82-12.9] | 63.58 | 514.59 [400.36-659.19] | 395.93 [307.58-509.18] | -0.94 [-1.03 to -0.85] |
| Cambodia | 81.09 [61.31-105.12] | 156.13 [118.47-201.64] | 92.54 | 266.95 [202.81-343.17] | 208.77 [158.13-268.64] | -0.9 [-0.98 to -0.83] |
| Cameroon | 162.39 [124.38-207.17] | 394.17 [297.83-504.85] | 142.73 | 535.85 [418.1-689.24] | 452.93 [346.22-585.35] | -0.5 [-0.59 to -0.4] |
| Canada | 530.44 [396.67-677.25] | 907.84 [682.02-1145.17] | 71.15 | 321.34 [241.91-412.4] | 297.65 [224.62-379.3] | -0.17 [-0.22 to -0.13] |
| Central African  Republic | 33.99 [25.93-43.87] | 60.26 [45.42-78.48] | 77.29 | 415.09 [319.94-533.6] | 373.16 [286.13-478.98] | -0.37 [-0.4 to -0.34] |
| Chad | 101.16 [78.69-128.07] | 208.84 [158.37-268.09] | 106.45 | 573.84 [448.92-730.2] | 533.62 [414.3-679.3] | -0.23 [-0.26 to -0.21] |
| Chile | 225.4 [172.68-287.92] | 374.19 [282.55-480.77] | 66.01 | 386.76 [296.37-492.24] | 311.79 [236.47-397.65] | -0.79 [-0.86 to -0.71] |
| China | 10514.87 [8678.93-  12653.63] | 19759.66 [16041.29-23739.46] | 87.92 | 228.66 [189.02-274.15] | 187.74 [154.21-224.43] | -0.26 [-0.44 to -0.08] |
| Colombia | 514.23 [397.66-658.04] | 1039.95 [799.99-1337.97] | 102.23 | 469.57 [363.31-598.83] | 368.67 [285.03-474.14] | -0.83 [-0.87 to -0.78] |
| Comoros | 5.4 [4.14-6.96] | 8.91 [6.78-11.56] | 65.00 | 395.8 [303.91-509.83] | 298.34 [226.58-385.65] | -1.03 [-1.06 to -0.99] |
| Congo | 29.14 [22.42-37.99] | 64.64 [49.32-85.61] | 121.83 | 410.25 [313.94-532.3] | 345.36 [263.65-445.48] | -0.48 [-0.54 to -0.43] |
| Costa Rica | 47.92 [37.55-60.86] | 94.8 [72.23-121.97] | 97.83 | 471.44 [366.01-601.51] | 340.13 [260.04-437.17] | -1.1 [-1.21 to -0.99] |
| Cote d'Ivoire | 163.47 [123.06-206.75] | 359.01 [273.26-457.14] | 119.62 | 550.18 [431.09-695.4] | 469.79 [366.64-598.94] | NA |
| Croatia | 80.72 [60.8-106.04] | 77.83 [61.44-97.64] | −3.58 | 236.6 [178.11-310.28] | 210.09 [168.54-262.88] | -0.39 [-0.63 to -0.14] |
| Cuba | 220.99 [171.4-277.72] | 293.02 [226.15-374] | 32.59 | 412.62 [318.78-520.34] | 360.14 [279.34-457.96] | -0.26 [-0.36 to -0.15] |
| Cyprus | 15.76 [12.02-20.16] | 24.78 [18.86-32.15] | 57.23 | 374.99 [287.67-478.88] | 268.53 [205.24-347.87] | -1.58 [-1.75 to -1.4] |
| Czechia | 204.82 [156.04-263.88] | 256.8 [193.72-329.98] | 25.38 | 299.38 [225.15-387.45] | 290.45 [218.31-379.27] | 0.46 [0.18 to 0.75] |
| Democratic  People's Republic  of Korea | 280.41 [213.28-366.32] | 343.08 [256.12-445.1] | 22.35 | 265.82 [203.99-341.41] | 189.68 [142.54-244.89] | -1.14 [-1.16 to -1.12] |
| Democratic  Republic of the  Congo | 478.46 [365.89-615.03] | 949.79 [725.71-1228.55] | 98.51 | 434.28 [335.91-560.12] | 374.75 [289.08-484.68] | -0.5 [-0.57 to -0.43] |
| Denmark | 139.92 [107.83-176.56] | 171.34 [130.97-221.39] | 22.46 | 403.91 [311.25-515.19] | 401.87 [310.12-513.03] | 0.1 [0.02 to 0.19] |
| Djibouti | 4.53 [3.41-5.84] | 13.98 [10.31-18.28] | 208.61 | 399.75 [308.66-508.35] | 321.6 [246.73-411.75] | -0.78 [-0.84 to -0.71] |
| Dominica | 1.58 [1.25-1.98] | 1.44 [1.12-1.83] | −8.86 | 483.24 [377.11-619.77] | 364.92 [281.74-462.6] | -1 [-1.03 to -0.96] |
| Dominican  Republic | 129.57 [100.36-163.72] | 218.89 [171.56-277.93] | 68.94 | 513.93 [401.05-656.34] | 419.06 [329.69-532.27] | -0.72 [-0.76 to -0.67] |
| Ecuador | 203.7 [163.91-247.83] | 368.91 [294.17-457.47] | 81.10 | 583.86 [474.17-709.62] | 440 [350.12-545.51] | -0.9 [-1.04 to -0.77] |
| Egypt | 917.5 [690.39-1214.26] | 1683.39 [1251.51-2247.2] | 83.48 | 475.74 [362.96-621.1 | 414 [314.37-544.11] | -0.37 [-0.41 to -0.33] |
| El Salvador | 85.25 [66.79-108.3] | 120.41 [92.17-154.71] | 41.24 | 487.05 [378-618.15] | 355.14 [270.8-456.72] | -1.08 [-1.2 to -0.96] |
|  |  |  |  |  |  |  |
| Equatorial Guinea | 6.08 [4.66-7.83] | 13.03 [10.04-17.01] | 114.31 | 445.15 [342.95-570.56] | 339.25 [260.68-435.4] | -0.99 [-1.05 to -0.92] |
| Eritrea | 32.43 [24.63-41.65] | 69.07 [52.53-88.81] | 112.98 | 390.77 [301.17-498.13] | 326.22 [250.77-422.44] | -0.67 [-0.71 to -0.62] |
| Estonia | 32.25 [23.86-41.64] | 31.02 [23.07-40.01] | −3.81 | 283.95 [212.71-367.19] | 268.55 [201.17-349.32] | 0.51 [0.17 to 0.85] |
| Eswatini | 8.61 [6.56-11.09] | 14.18 [10.95-18.08] | 64.69 | 398.94 [308.58-513.85] | 345.37 [266.74-441.36] | -0.46 [-0.5 to -0.43] |
| Ethiopia | 582.15 [477.51-703.44] | 1106.75 [912.35-1328.01] | 90.11 | 428.56 [351.3-516.29] | 374.84 [309.52-448.65] | -0.51 [-0.54 to -0.48] |
| Fiji | 5.31 [4.04-7] | 9.16 [6.99-11.95] | 72.50 | 224.55 [171.68-290.23] | 212.1 [163.01-272.07] | -0.18 [-0.21 to -0.16] |
| Finland | 136.22 [105.24-176.5] | 155.94 [118.47-204.19] | 14.48 | 396.19 [305.82-512.53] | 362.52 [278.13-474.24] | -0.31 [-0.39 to -0.22] |
| France | 1389.42 [1057.66-1783] | 1875.09 [1448.04-2394.6] | 34.95 | 374 [288.41-482.03] | 373.53 [290.77-477.27] | 0.23 [0.14 to 0.31] |
| Gabon | 13.19 [10.27-16.59] | 22.44 [17.19-29.04] | 70.13 | 409.82 [317.68-522.28] | 323.48 [250.42-415.67] | -0.83 [-0.85 to -0.82] |
| Gambia | 13.38 [10.2-17.01] | 29.69 [23.2-37.86] | 121.90 | 564.05 [442.89-716.31] | 452.26 [354.84-574.68] | -0.77 [-0.83 to -0.71] |
| Germany | 2279.42 [1764.32-2946.46] | 2635.36 [1998.83-3395.62] | 15.62 | 394.65 [306.85-508.47] | 388.13 [298.67-496.77] | 0.22 [0.08 to 0.36] |
| Ghana | 232.28 [176.8-294.15] | 508.74 [391.08-655.36] | 119.02 | 527.27 [412.35-674.36] | 427.8 [329.34-547.07] | -0.67 [-0.89 to -0.45] |
| Greece | 222.09 [167.79-291.25] | 273.91 [212.13-352.65] | 23.33 | 319.34 [243.16-415.51] | 319.7 [245.67-415.91] | 0.29 [0.15 to 0.43] |
| Greenland | 0.67 [0.5-0.86] | 1.04 [0.79-1.34] | 55.22 | 349.06 [259.13-445.37] | 313.41 [236.87-398.62] | -0.41 [-0.49 to -0.33] |
| Grenada | 1.71 [1.34-2.16] | 2.13 [1.66-2.76] | 24.56 | 494.16 [387.28-629.76] | 373.3 [290.98-479.33] | -0.89 [-0.96 to -0.81] |
| Guam | 1.09 [0.82-1.43] | 2.18 [1.65-2.8] | 100.00 | 238.41 [180.91-310.1] | 229.64 [174.78-295.2] | -0.18 [-0.25 to -0.11] |
| Guatemala | 127.6 [98.78-162.96] | 256.29 [199.13-327.76] | 100.85 | 554.52 [426.55-703.48] | 371.55 [285.64-474.56] | -1.44 [-1.54 to -1.34] |
| Guinea | 111.9 [86.43-140.89] | 175.38 [134.51-223.95] | 56.73 | 562.34 [437.81-716.1] | 472.74 [368.09-600.48] | -0.61 [-0.63 to -0.59] |
| Guinea-Bissau | 15.78 [12.2-20.03] | 26.57 [20.12-33.77] | 68.38 | 548.67 [431.19-689.88] | 463.01 [361.6-591.25] | -0.58 [-0.63 to -0.54] |
| Guyana | 12.64 [9.79-16] | 14.03 [10.91-17.94] | 11.00 | 470.22 [364.6-599.57] | 373.26 [290.4-476.35] | -0.7 [-0.75 to -0.65] |
| Haiti | 120.11 [92.42-152.05] | 217.96 [166.67-279.86] | 81.47 | 542.37 [425.42-683.45] | 420.23 [329.43-535.97] | -0.84 [-0.87 to -0.81] |
| Honduras | 69.11 [53.52-87.37] | 144.34 [111.15-186.7] | 108.86 | 543.35 [420.5-684.95] | 384.98 [297.09-495.64] | -1.22 [-1.31 to -1.13] |
| Hungary | 220.01 [165.19-284.08] | 222.22 [165.47-287.56] | 1.00 | 297.78 [220.94-388] | 259.76 [194.69-343.51] | -0.25 [-0.45 to -0.05] |
| Iceland | 6.28 [4.84-7.96] | 9.73 [7.42-12.52] | 54.94 | 474.9 [366.49-603.49] | 431.22 [332.16-552.65] | 0.27 [0.04 to 0.51] |
| India | 18859.45 [15367.9-  22403.29] | 26383.41 [21589.21-31565.13] | 39.89 | 611.36 [502.93-736.58] | 400.06 [325.98-476.91] | -1.38 [-1.43 to -1.32] |
| Indonesia | 1388.95 [1144.1-1674.37] | 2537.78 [2074.78-3051.19] | 82.71 | 226.5 [186.77-271.19] | 194.28 [159.8-231.97] | -0.48 [-0.54 to -0.42] |
| Iran (Islamic  Republic of) | 956.38 [775.35-1168.63] | 2266.78 [1824.84-2804.52] | 137.02 | 575.9 [470.88-700.53] | 477.39 [387.03-588.35] | -0.42 [-0.59 to -0.26] |
| Iraq | 255.24 [190.92-330.51] | 651.96 [489.13-860.44] | 155.43 | 509.5 [392.48-654.03] | 401.85 [307.36-522.24] | -0.86 [-0.92 to -0.81] |
| Ireland | 73.11 [56.36-93.53] | 122.4 [93.05-156.47] | 67.42 | 386.79 [299.72-498.05] | 368.09 [281.44-470.49] | 0.09 [-0.02 to 0.19] |
| Israel | 104.75 [81.07-135.69] | 227.88 [177.12-291.37] | 117.55 | 433.21 [332.1-559.35] | 438.92 [341.3-560.33] | 0.19 [0.09 to 0.3] |
| Italy | 1189.96 [950.03-1455.96] | 1377.64 [1112.75-1646.65] | 15.77 | 295.02 [235.65-361.04] | 263.73 [214.94-315.34] | -0.55 [-0.85 to -0.24] |
| Jamaica | 42.86 [33.45-53.07] | 1377.64 [1112.75-1646.65] | 23.85 | 464.07 [357.65-584.74] | 344.46 [266.07-439.71] | -1.04 [-1.08 to -1.01] |
| Japan | 1411.06 [1125.83-1735.17] | 1918.5 [1527.15-2332.75] | 35.96 | 153.01 [122.32-187.39] | 133.29 [107.24-161.06] | -0.45 [-0.55 to -0.35] |
| Jordan | 39.33 [29.3-51.36] | 148.97 [112.58-195.54] | 278.77 | 415.94 [318.53-538.4] | 332.66 [256.05-430.66] | -0.6 [-0.71 to -0.5] |
| Kazakhstan | 171.13 [127.08-226.55] | 223.8 [168.08-294.38] | 30.78 | 225.07 [167.24-297.59] | 212.21 [160.66-278.12] | 0.3 [-0.01 to 0.6] |
| Kenya | 235.65 [196.01-281.73] | 523.15 [432.47-629.07] | 122.00 | 417.34 [345.59-500.88] | 323.36 [267.44-386.04] | -0.87 [-0.9 to -0.84] |
| Kuwait | 16.83 [12.04-22.24] | 70.1 [50.27-93.77] | 316.52 | 354.02 [269.86-459.37] | 288.28 [221.28-375.91] | -1.13 [-1.48 to -0.77] |
| Kyrgyzstan | 41.29 [30.81-53.34] | 62.09 [46.43-82.29] | 50.38 | 247.67 [185.59-322.31] | 213.68 [162.27-280.96] | 0.01 [-0.29 to 0.31] |
| Lao People's  Democratic  Republic | 33.51 [25.62-43.92] | 59.21 [45.02-76.63] | 76.69 | 266.98 [205.1-344.07] | 218.52 [166.42-279.1] | -0.86 [-0.92 to -0.8] |
| Latvia | 53 [39.39-68.54] | 46.32 [34.24-59.7] | −12.60 | 264.52 [198.75-341.96] | 257.79 [193.19-331.81] | 0.2 [-0.2 to 0.59] |
| Lebanon | 61.81 [47.71-80.97] | 112.2 [85.99-147.49] | 81.52 | 469.05 [364.76-611.37] | 383.84 [295.32-500.88] | -0.67 [-0.69 to -0.65] |
| Lesotho | 22.97 [17.73-29.38] | 27.76 [21.4-35.22] | 20.85 | 388.05 [299.43-502.29] | 332.26 [255.92-423.66] | -0.5 [-0.54 to -0.45] |
| Liberia | 33.9 [26.28-42.95] | 67.77 [51-88.47] | 99.91 | 556.94 [435.71-710.26] | 447.15 [346.76-579.01] | -0.76 [-0.82 to -0.7] |
| Libya | 49.33 [36.7-63.38] | 121.82 [90.6-161.75] | 146.95 | 460.35 [346.21-595.13] | 336.09 [255.49-438.58] | -0.89 [-0.98 to -0.79] |
| Lithuania | 73.47 [54.71-94.29] | 73.47 [55.43-95.38] | 0.00 | 294.09 [218.9-381.86] | 281.05 [211.8-367.85] | 0.09 [-0.17 to 0.34] |
| Luxembourg | 9.02 [6.93-11.75] | 14.38 [11.22-18.61] | 59.42 | 347.62 [266.46-449.67] | 328.37 [254.96-423.71] | -0.64 [-0.79 to -0.49] |
| Madagascar | 128.7 [97.97-164.84] | 278.52 [212.2-358.82] | 116.41 | 389.64 [297.41-499.74] | 335.07 [261.67-428.48] | -0.53 [-0.58 to -0.48] |
| Malawi | 108.8 [83.49-139.84] | 186.47 [142.29-237.45] | 71.39 | 406.69 [314.01-524.94] | 350.4 [269.47-447.19] | -0.54 [-0.6 to -0.47] |
| Malaysia | 129.94 [99.02-169.29] | 288.65 [220.93-374.57] | 122.14 | 230.52 [172.72-295.83] | 198.83 [152.15-257.76] | -0.65 [-0.72 to -0.59] |
| Maldives | 1.26 [0.95-1.65] | 3.14 [2.38-4.11] | 149.21 | 251.32 [191.17-324.05] | 184.98 [139.5-236.14] | -0.96 [-1.07 to -0.85] |
| Mali | 153.11 [118.6-195.16] | 301.3 [231.28-387.36] | 96.79 | 580.07 [456.46-736.17] | 521.57 [406.38-666.58] | -0.38 [-0.4 to -0.36] |
| Malta | 8.06 [6.16-10.43] | 11.09 [8.32-14.3] | 37.59 | 358.42 [274.17-462.61] | 322.71 [246.4-415.19] | -0.44 [-0.81 to -0.08] |
| Marshall Islands | 0.25 [0.19-0.32] | 0.47 [0.36-0.62] | 88.00 | 254.72 [195.61-328.52] | 219.43 [165.9-285.33] | -0.44 [-0.47 to -0.41] |
| Mauritania | 33.67 [26.21-42.7] | 58.53 [45.55-75.27] | 73.83 | 542.03 [423.64-693.91] | 439.26 [343.56-564.6] | -0.68 [-0.74 to -0.62] |
| Mauritius | 8.76 [6.65-11.23] | 15.54 [11.67-20.14] | 77.40 | 202.1 [153.1-259.62] | 170.74 [129.82-221.79] | -0.63 [-0.67 to -0.6] |
| Mexico | 1260.45 [1036.12-1529.44] | 2837.58 [2324.29-3402.83] | 125.12 | 475 [387.01-573.14] | 423.23 [347.96-505.63] | -0.82 [-0.97 to -0.67] |
| Micronesia  (Federated States  of) | 0.69 [0.53-0.88] | 0.89 [0.66-1.14] | 28.99 | 262.76 [201.19-334.33] | 207.35 [157.68-265.37] | -0.81 [-0.84 to -0.78] |
| Mongolia | 15.42 [11.55-20.33] | 37.88 [27.63-51.6] | 145.65 | 264.66 [199.9-347.55] | 230.35 [171.87-303.5] | 0.01 [-0.32 to 0.35] |
| Montenegro | 10.18 [7.71-13.34] | 12.3 [9.25-16.11] | 20.83 | 297.46 [226.31-387.16] | 263.23 [198.37-346.57] | -0.37 [-0.48 to -0.27] |
| Morocco | 394.02 [297.76-509.13] | 726.94 [549.24-938.14] | 84.49 | 450.28 [347.69-579.6] | 390.18 [295.98-500.6] | -0.48 [-0.53 to -0.44] |
| Mozambique | 162.99 [123.5-210.02] | 298.5 [227.01-385.34] | 83.14 | 389.54 [300.75-500.02] | 358.81 [275.96-461.38] | -0.28 [-0.32 to -0.24] |
| Myanmar | 329.09 [249.72-425.45] | 584.33 [439.83-758.76] | 77.56 | 240.5 [182.59-309.7] | 204.13 [154.47-263.58] | -0.54 [-0.57 to -0.51] |
| Namibia | 17 [13.18-21.72] | 33.32 [25.84-42.77] | 96.00 | 393.71 [305-507] | 354.02 [275.21-453.65] | -0.31 [-0.34 to -0.29] |
| Nepal | 580.73 [453.73-724.1] | 724.89 [573.82-890.24] | 24.82 | 851.27 [679.53-1057.75] | 502.65 [397.55-617.94] | -1.84 [-2.11 to -1.58] |
| Netherlands | 341.41 [263.09-441.01] | 465.56 [359.9-601.7] | 36.36 | 363.76 [279.64-472.83] | 357.85 [279.36-461.82] | 0.2 [0.11 to 0.3] |
| New Zealand | 69.76 [56.32-84.01] | 121.7 [97.49-147.89] | 74.46 | 364.85 [295.37-444.12] | 353.12 [288.77-427.74] | -0.28 [-0.75 to 0.2] |
| Nicaragua | 52.93 [41.18-67.7] | 104.91 [81.22-135.89] | 98.21 | 530.31 [408.46-676.4] | 373.64 [288.78-476.8] | -1.17 [-1.28 to -1.06] |
| Niger | 118.62 [90.42-152.07] | 315.45 [243.1-400.63] | 165.93 | 591.92 [465.57-752.18] | 559.5 [438.37-710.2] | -0.2 [-0.22 to -0.18] |
| Nigeria | 1470.91 [1219.58-1758.19] | 3313.75 [2730.51-4009.2] | 125.29 | 600.75 [496.67-724.66] | 495.04 [410.07-595] | -0.66 [-0.72 to -0.61] |
| North Macedonia | 30.84 [23.3-40.1] | 38.2 [28.36-49.44] | 23.87 | 298.54 [226.43-384.28] | 244.14 [182.36-318.45] | -0.62 [-0.8 to -0.43] |
| Northern Mariana  Islands | 0.25 [0.19-0.33] | 0.58 [0.43-0.76] | 132.00 | 216.58 [164.39-281.18] | 198.69 [151.33-255.42] | -0.25 [-0.28 to -0.22] |
| Norway | 128.87 [103.77-154.5] | 168.05 [135.11-200.73] | 30.40 | 472.1 [384.51-564.44] | 453 [370.63-542.23] | -0.26 [-0.33 to -0.19] |
| Oman | 22.72 [16.97-30.24] | 50.11 [36.15-66.89] | 120.55 | 521.67 [404.17-675.07] | 359.25 [273.87-468.27] | -1.42 [-1.47 to -1.37] |
| Pakistan | 2868.48 [2322.38-3459.43] | 4608.04 [3741.86-5532.35] | 60.64 | 830.44 [677.73-1005.32] | 575.88 [470.21-690.34] | -1.25 [-1.33 to -1.16] |
| Palestine | 29.55 [22.53-38.32] | 72.85 [53.86-95.77] | 146.53 | 527.51 [405.37-683.49] | 420.34 [319.6-545.4] | -0.84 [-0.95 to -0.73] |
| Panama | 38.41 [29.7-49.1] | 79.99 [60.94-102.87] | 108.25 | 461.05 [353.33-588.51] | 373.96 [285.18-479.2] | -0.66 [-0.74 to -0.57] |
| Papua New Guinea | 28.71 [21.85-37.37] | 75.43 [56.87-98.47] | 162.73 | 258.01 [194.24-332.17] | 247.38 [185.75-318.6] | -0.15 [-0.16 to -0.15] |
| Paraguay | 87.45 [66.93-110.41] | 147.73 [113.67-182.07] | 68.93 | 568.92 [441.22-707.71] | 445.57 [345.78-574.44] | -0.77 [-0.83 to -0.71] |
| Peru | 420.78 [328.85-533.28] | 781.03 [604.34-1008.34] | 85.61 | 558.31 [435.48-711.54] | 445.57 [345.78-574.44] | -0.75 [-0.8 to -0.7] |
| Philippines | 470.46 [387.29-563.13] | 1047.31 [860.86-1261.1] | 122.61 | 253.87 [209.13-304.26] | 225.67 [185.86-269.7] | -0.4 [-0.43 to -0.38] |
| Poland | 749.14 [595.95-919.24] | 1078.36 [866.37-1305.76] | 43.95 | 334.58 [269.22-412.68] | 352.42 [286.37-429.75] | 0.5 [-0.1 to 1.1] |
| Portugal | 187.07 [144.03-242.69] | 210.73 [161.56-268.3] | 12.65 | 280.49 [215.93-362.95] | 226.6 [174.04-289.59] | -0.72 [-0.87 to -0.56] |
| Puerto Rico | 86.2 [66.36-109.23] | 86.92 [66.61-110.88] | 0.84 | 452.05 [350.09-571.79] | 322.84 [248.23-410.94] | -1.14 [-1.26 to -1.02] |
| Qatar | 4.51 [3.11-6.08] | 25.53 [17.58-34.98] | 466.08 | 445.41 [338.47-575.77] | 339.33 [257.64-440.96] | -0.95 [-0.98 to -0.92] |
| Republic of Korea | 321.45 [240.1-421.18] | 676.07 [504.39-884.19] | 110.32 | 174.27 [130.44-229.41] | 146.01 [109.28-188.73] | -0.75 [-0.95 to -0.54] |
| Republic of  Moldova | 76.63 [57.22-100.13] | 72.34 [53.32-93.49] | −5.60 | 301.39 [227.17-393.6] | 241.45 [180.11-316.93] | -0.3 [-0.62 to 0.03] |
| Romania | 350.65 [255.76-454.21] | 390.03 [291.19-505.23] | 11.23 | 241.36 [177.03-313.71] | 236.4 [178.74-310.57] | 0.15 [-0.07 to 0.37] |
| Russian Federation | 2903.34 [2330.81-3497.98] | 3454.54 [2744.91-4145.94] | 18.99 | 279.48 [228.73-336.42] | 280.65 [228.96-338.07] | 0.8 [0.35 to 1.26] |
| Rwanda | 79.9 [61.71-103.18] | 146.5 [111.31-189.83] | 83.35 | 395.34 [303.76-512.2] | 326.6 [249.63-419.01] | -0.76 [-0.79 to -0.72] |
| Saint Lucia | 2.39 [1.87-3.02] | 3.62 [2.79-4.7] | 51.46 | 471.08 [364.99-601.24] | 329.66 [254.17-420.03] | -1.19 [-1.25 to -1.14] |
| Saint Vincent and  the Grenadines | 1.9 [1.49-2.33] | 2.4 [1.86-3.07] | 26.32 | 483.76 [379.38-616.18] | 376.7 [291.35-479.72] | -0.82 [-0.86 to -0.78] |
| Samoa | 1 [0.76-1.29] | 1.28 [0.97-1.67] | 28.00 | 209.22 [158.9-271.01] | 160.39 [123.21-208.22] | -0.94 [-0.98 to -0.89] |
| Sao Tome and  Principe | 1.98 [1.56-2.49] | 3.1 [2.35-4.07] | 56.57 | 548.91 [429.8-705.53] | 427.28 [327.11-551.48] | -0.88 [-0.95 to -0.81] |
| Saudi Arabia | 186.68 [137.89-245.8] | 544.04 [385.96-715.04] | 191.43 | 488.82 [375.43-631.5] | 348.69 [262.53-450.09] | -1.26 [-1.31 to -1.2] |
| Senegal | 115.64 [88.62-144.76] | 231.52 [178.27-299.2] | 100.21 | 550.86 [429.48-693.95] | 464.8 [359.95-597.6] | -0.53 [-0.57 to -0.49] |
| Serbia | 155.07 [115.45-205.01] | 147.11 [109.75-191.58] | −5.13 | 252.46 [190.69-330.59] | 214.57 [161.18-281.86] | -0.6 [-0.8 to -0.4] |
| Seychelles | 0.63 [0.49-0.8] | 1.24 [0.93-1.6] | 96.83 | 216.54 [166.28-277.64] | 206.1 [157.06-265.51] | -0.08 [-0.15 to 0] |
| Sierra Leone | 61.28 [47.74-78.42] | 114.01 [87.85-145.37] | 86.05 | 535.79 [421.19-680.35] | 464.94 [366.81-594.66] | -0.49 [-0.54 to -0.44] |
| Singapore | 21.88 [16.24-28.41] | 57.7 [43.32-74.85] | 163.71 | 168.01 [125.02-219.71] | 143.16 [107.94-184.23] | -0.52 [-0.69 to -0.35] |
| Slovakia | 98.91 [75.52-127.59] | 121.86 [91.99-158.88] | 23.20 | 326.44 [249.31-427.9] | 281.57 [213.29-373.09] | -0.24 [-0.53 to 0.05] |
| Slovenia | 39.42 [30.08-51.25] | 53.29 [40.33-68.24] | 35.19 | 308.76 [234.33-406.05] | 306.1 [231.62-398.57] | 0.32 [0.14 to 0.5] |
| Solomon Islands | 2.24 [1.69-2.92] | 5.01 [3.78-6.62] | 123.66 | 284.43 [217.94-368.74] | 248.16 [189.39-320.15] | -0.49 [-0.52 to -0.46] |
| Somalia | 83.42 [63.02-107.54] | 201.18 [153.86-254.85] | 141.17 | 407.99 [317.42-517.88] | 375.36 [294.2-477.25] | -0.31 [-0.34 to -0.29] |
| South Africa | 497.33 [413.27-595.6] | 897.09 [740.77-1075.93] | 80.38 | 371.18 [305.3-444.13] | 321.61 [264.78-384.86] | -0.52 [-0.54 to -0.5] |
| South Sudan | 53.78 [41.23-68.44] | 99.97 [75.19-128.48] | 85.89 | 381.58 [293.85-489.6] | 356.92 [274.95-451.57] | -0.24 [-0.27 to -0.21] |
| Spain | 824.71 [637.62-1064.98] | 1215.54 [937.44-1569.61] | 47.39 | 331.49 [256.73-427.45] | 324.76 [251.18-420.02] | 0.22 [0.09 to 0.34] |
| Sri Lanka | 132.38 [101.74-172.65] | 265.02 [198.25-347.12] | 100.20 | 208.6 [159.71-269.74] | 191.17 [144.95-249.21] | -0.23 [-0.31 to -0.16] |
| Sudan | 312.38 [234.94-403.29] | 594.07 [425.71-774.9] | 90.18 | 518.34 [398.67-665.41] | 420.65 [317.43-539.29] | -0.74 [-0.81 to -0.67] |
| Suriname | 7.3 [5.65-9.24] | 12.28 [9.48-15.65] | 68.22 | 469.73 [363.62-594.58] | 380.33 [295.01-480.27] | -0.62 [-0.69 to -0.56] |
| Sweden | 206.75 [167.66-246.27] | 217.9 [175.4-262.82] | 5.39 | 354.12 [291.53-419.08] | 284.15 [233.41-341.68] | -0.65 [-0.82 to -0.48] |
| Switzerland | 175.47 [134.26-224.84] | 243.57 [186.59-317.61] | 38.81 | 378.01 [286.96-485.53] | 364.78 [277.58-470.46] | -0.1 [-0.33 to 0.12] |
| Syrian Arab  Republic | 162.26 [121.32-209.62] | 274.65 [204.5-363.99] | 69.27 | 486.53 [374.23-625.86] | 363.89 [275.14-475.68] | -0.81 [-1 to -0.63] |
| Tajikistan | 41.21 [31.15-52.81] | 78.9 [57.1-106.37] | 91.46 | 274.75 [206.47-352.99] | 230.5 [170.52-300.93] | -0.38 [-0.6 to -0.15] |
| Thailand | 452.64 [345.88-584.19] | 905.37 [681.31-1186.6] | 100.02 | 203.9 [156.45-262.69] | 166.16 [125.96-214.97] | -0.69 [-0.73 to -0.65] |
| Timor-Leste | 5.59 [4.21-7.47] | 10.29 [7.9-13.3] | 84.08 | 280.92 [216.83-367.42] | 236.89 [183.01-303.28] | -0.65 [-0.73 to -0.57] |
| Togo | 53.33 [40.73-67.89] | 128.84 [98.71-166.7] | 141.59 | 546.25 [426.3-699.46] | 452.64 [350.46-580.29] | -0.58 [-0.64 to -0.52] |
| Tonga | 0.76 [0.57-0.99] | 0.96 [0.73-1.24] | 26.32 | 238.77 [180.32-307.33] | 220.19 [167.54-283.32] | -0.24 [-0.27 to -0.21] |
| Trinidad and  Tobago | 21.49 [16.7-27.7] | 29.52 [23.06-38] | 37.37 | 430.86 [336.5-553.1] | 337.15 [262.99-430.2] | -0.63 [-0.73 to -0.54] |
| Tunisia | 127.78 [95.51-166.05] | 246.56 [186.49-321.82] | 92.96 | 437.72 [335.81-562.94] | 356.27 [271.01-462.85] | -0.68 [-0.71 to -0.64] |
| Turkey | 887.53 [670.33-1166.19] | 1678.2 [1287.91-2182.48] | 89.09 | 402.78 [308.65-531.17] | 349.11 [267.91-453] | -0.43 [-0.46 to -0.4] |
| Turkmenistan | 29.08 [21.94-38.46] | 54.19 [40.05-72.03] | 86.35 | 264.26 [198.53-350.08] | 222.31 [166.94-293.31] | -0.29 [-0.58 to 0.01] |
| Uganda | 178.8 [137.1-226.94] | 401.97 [304.34-512.29] | 124.82 | 412.17 [317.04-525.52] | 366.43 [281.18-468.26] | -0.3 [-0.45 to -0.15] |
| Ukraine | 1162.66 [930.27-1423.31] | 1012.62 [811.32-1216.3] | −12.90 | 291.48 [237.03-352.04] | 256.37 [208.33-308.93] | 0.23 [-0.18 to 0.64] |
| United Arab  Emirates | 16.2 [11.31-21.79] | 104.55 [71.36-143.41] | 545.37 | 434.15 [330.81-565.63] | 314.05 [238.66-412.94] | -1.01 [-1.07 to -0.96] |
| United Kingdom | 1550.2 [1239.52-1877.11] | 1887.9 [1535.99-2252.85] | 21.78 | 400.98 [324.3-485.66]3 | 396.24 [326.55-472.46] | 0.25 [0.11 to 0.4] |
| United Republic of  Tanzania | 285.05 [220.88-362.96] | 623.5 [479.95-791.69] | 118.73 | 396.08 [308.27-508.25] | 354.98 [275.33-455.06] | -0.37 [-0.4 to -0.33] |
| United States of  America | 5413.84 [4368.99-6436.89] | 8030.83 [6580.88-9470.96] | 48.34 | 346.37 [279.62-419.52] | 309.33 [257.02-365.19] | -0.62 [-0.68 to -0.56] |
| United States Virgin  Islands | 2.68 [2.06-3.48] | 2.84 [2.17-3.65] | 5.97 | 487.74 [378.72-622.33] | 375.55 [289.76-482.68] | -0.91 [-0.95 to -0.86] |
| Uruguay | 71.67 [54.22-91.42] | 77.11 [59.32-97.57] | 7.59 | 385.78 [292.09-497.14] | 326.34 [250.21-416.94] | -0.48 [-0.6 to -0.36] |
| Uzbekistan | 169.59 [126.25-221.44] | 318.74 [233.57-429.91] | 87.95 | 268.81 [200.51-350.72] | 218.31 [165.85-288.14] | -0.46 [-0.73 to -0.19] |
| Vanuatu | 0.98 [0.75-1.27] | 2.18 [1.67-2.86] | 122.45 | 263.23 [200.67-338.43] | 227.13 [171.75-294.92] | -0.54 [-0.56 to -0.52] |
| Venezuela  (Bolivarian Republic  of) | 296.01 [230.14-378.81] | 583.78 [447.58-749.54] | 97.22 | 486.3 [377.71-622.93] | 364.55 [280.27-466.21] | -0.8 [-0.88 to -0.72] |
| Viet Nam | 531.04 [410.86-680.35] | 1040.88 [785.4-1352.92] | 96.01 | 227.44 [173.05-294.35] | 185.24 [140.44-238.9] | -0.64 [-0.69 to -0.6] |
| Yemen | 188.56 [140.81-243.11] | 474.2 [348.22-623.67] | 151.48 | 537.46 [419.98-682.1] | 447.63 [342.77-585.25] | -0.65 [-0.73 to -0.56] |
| Zambia | 79.75 [60.77-101.97] | 177.45 [135.15-226.99] | 122.51 | 400.68 [312.37-510.26] | 348.52 [268.79-447.82] | -0.49 [-0.54 to -0.45] |
| Zimbabwe | 108.83 [84.19-139.38] | 187.33 [144.06-240.32] | 72.13 | 407.98 [315.71-523.11] | 368.46 [284.92-468.73] | -0.25 [-0.3 to -0.2] |
