# Supplementary material for: Global burden and trends of pelvic organ prolapse associated with aging women: An observational trend study from 1990 to 2019
Source: Front Public Health. 2022 Sep 15;10:975829. doi: 10.3389/fpubh.2022.975829 (PMC9521163; doi:10.3389/fpubh.2022.975829)
Supplement: Supplementary Table 2 — The DALY rate and age standardized DALY rate of POP and the trend of time changes from 1990 to 2019. [file Data_Sheet_2.docx]

Supplementary Table2. The DALY rate and age standardized DALY rate of POP and the trend of time changes from 1990 to 2019

| Nation | DALY No.(95% UI) | | Rate of change (%) | Age Standardized DALY Rate No.(95% UI) | | 1990-2019  EAPC No. (95% CI) |
| --- | --- | --- | --- | --- | --- | --- |
| Afghanistan | 6.29 [2.94-12.12] | 14.47 [6.91-28.43] | 130.05 | 16.16 [7.57-30.91] | 15.78 [7.48-30.66] | -0.08 [-0.14 to -0.01] |
| Albania | 1.55 [0.9-2.82] | 2.17 [1.24-3.74] | 40.00 | 13.14 [7.33-24.17] | 10.76 [6.4-18.45] | -0.81 [-0.91 to -0.71] |
| Algeria | 10.09 [4.79-19.25] | 26.06 [11.96-50.39] | 158.28 | 14.32 [6.76-27.22] | 13.3 [6.14-25.09] | -0.02 [-0.27 to 0.23] |
| American Samoa | 0.01 [0.01-0.03] | 0.02 [0.01-0.04] | 100.00 | 10.15 [5.9-17.65] | 8.09 [4.56-13.69] | -0.57 [-0.78 to -0.36] |
| Andorra | 0.03 [0.01-0.05] | 0.06 [0.03-0.12] | 100.00 | 9.89 [4.98-18.6] | 9.45 [4.6-18] | -0.02 [-0.08 to 0.04] |
| Angola | 3.79 [1.95-6.75] | 10.43 [5.61-18.84] | 175.20 | 16.09 [8.47-28.8] | 13.49 [7.16-24.47] | -0.62 [-0.69 to -0.55] |
| Antigua and  Barbuda | 0.04 [0.02-0.07] | 0.06 [0.03-0.11] | 50.00 | 13.22 [6.26-25.37] | 10.21 [4.84-19.73] | -0.96 [-0.99 to -0.93] |
| Argentina | 21.25 [10.49-40.57] | 27.29 [13.05-51.49] | 28.42 | 12.18 [6.01-23.3] | 9.82 [4.65-18.53] | -0.59 [-0.67 to -0.52] |
| Armenia | 1.01 [0.5-1.87] | 1.24 [0.66-2.25] | 22.77 | 6.35 [3.2-11.68] | 5.26 [2.77-9.48] | -0.28 [-0.63 to 0.07] |
| Australia | 7.25 [3.64-13.36] | 12.77 [6.04-24.62] | 76.14 | 7.16 [3.54-13.32] | 6.47 [3.02-12.31] | -0.1 [-0.23 to 0.03] |
| Austria | 6.63 [3.32-12.46] | 6.9 [3.31-12.85] | 4.07 | 11.2 [5.54-21.38] | 8.56 [4.06-16.16] | -1.26 [-1.53 to -1] |
| Azerbaijan | 2.75 [1.51-4.58] | 3.96 [2.24-6.75] | 44.00 | 9.18 [4.97-15.25] | 7.02 [3.93-12.08] | -0.62 [-0.83 to -0.4] |
| Bahamas | 0.13 [0.06-0.26] | 0.23 [0.11-0.44] | 76.92 | 13.84 [6.6-26.33] | 10.06 [4.85-19.15] | -1.2 [-1.26 to -1.14] |
| Bahrain | 0.13 [0.06-0.27] | 0.61 [0.28-1.21] | 369.23 | 12.25 [5.88-23.95] | 10.25 [4.78-19.85] | -0.6 [-0.67 to -0.52] |
| Bangladesh | 92.99 [53.41-157.65] | 153.03 [84.92-269.99] | 64.57 | 31.6 [17.88-53.79] | 20.7 [11.46-35.22] | -1.36 [-1.44 to -1.29] |
| Barbados | 0.19 [0.1-0.35] | 0.25 [0.13-0.47] | 31.58 | 13.6 [7.05-25.05] | 10.55 [5.4-19.78] | -0.85 [-0.95 to -0.74] |
| Belarus | 5.98 [2.84-11.41] | 6.86 [3.14-12.73] | 14.72 | 7.53 [3.55-14.22] | 7.44 [3.42-14.01] | 0.64 [0.24 to 1.04] |
| Belgium | 7.44 [3.52-14.34] | 9.7 [4.61-18.32] | 30.38 | 10.02 [4.65-19.24] | 10.07 [4.87-19.06] | -0.05 [-0.14 to 0.04] |
| Belize | 0.09 [0.05-0.18] | 0.21 [0.11-0.38] | 133.33 | 19.01 [9.77-35.41] | 12.54 [6.45-23.13] | -1.43 [-1.5 to -1.36] |
| Benin | 2.33 [1.2-4.22] | 4.99 [2.44-9.22] | 114.16 | 18.76 [9.44-35.14] | 15.81 [7.54-29.71] | -0.58 [-0.61 to -0.55] |
| Bermuda | 0.05 [0.03-0.09] | 0.06 [0.03-0.11] | 20.00 | 13.61 [7.31-24.95] | 10.56 [5.37-19.61] | -0.82 [-0.92 to -0.72] |
| Bhutan | 0.54 [0.29-0.94] | 0.73 [0.4-1.27] | 35.19 | 32.52 [17.82-57.46] | 22.9 [12.57-39.05] | -1.2 [-1.22 to -1.19] |
| Bolivia  (Plurinational State  of) | 4.24 [2.24-7.75] | 8.43 [4.3-15.33] | 98.82 | 21.5 [11.29-39.26] | 16.88 [8.58-30.85] | -0.8 [-0.91 to -0.69] |
| Bosnia and  Herzegovina | 2.02 [1.02-3.73] | 2.08 [1.03-3.87] | 2.97 | 8.18 [4.19-14.92] | 6.8 [3.45-12.38] | -0.58 [-0.84 to -0.32] |
| Botswana | 0.4 [0.2-0.75] | 0.85 [0.42-1.57] | 112.50 | 11.31 [5.51-20.94] | 9.41 [4.6-17.29] | -0.63 [-0.66 to -0.59] |
| Brazil | 98.11 [47.75-179.36] | 156.77 [77.94-288.58] | 59.79 | 16.27 [7.99-29.33] | 12.1 [6.03-22.24] | -1.31 [-1.43 to -1.19] |
| Brunei Darussalam | 0.04 [0.02-0.06] | 0.09 [0.05-0.15] | 125.00 | 6.09 [3.15-11.13] | 4.82 [2.65-8.59] | -0.8 [-0.91 to -0.69] |
| Bulgaria | 5.01 [2.31-9.47] | 5.06 [2.35-9.86] | 1.00 | 7.55 [3.45-14.26] | 7.07 [3.22-13.51] | 0.17 [-0.04 to 0.38] |
| Burkina Faso | 5.16 [2.63-9.52] | 10.16 [4.92-18.96] | 96.90 | 19.07 [9.58-35.33] | 16.64 [7.93-31.39] | -0.55 [-0.59 to -0.51] |
| Burundi | 1.9 [0.98-3.5] | 3.37 [1.75-6.13] | 77.37 | 13.09 [6.67-24.36] | 12.03 [6.1-22.24] | -0.26 [-0.29 to -0.24] |
| Cabo Verde | 0.2 [0.09-0.38] | 0.29 [0.13-0.55] | 45.00 | 15.88 [7.29-30.48] | 11.64 [5.4-22.33] | -1.13 [-1.24 to -1.02] |
| Cambodia | 2.34 [1.2-4.49] | 4.98 [2.7-8.92] | 112.82 | 8.09 [4.03-15.31] | 6.71 [3.61-12.07] | -0.6 [-0.74 to -0.46] |
| Cameroon | 5.05 [2.62-9.14] | 10.72 [5.2-20.02] | 112.28 | 17.88 [8.96-33.24] | 14.03 [6.7-26.58] | -0.76 [-0.86 to -0.67] |
| Canada | 14.22 [6.7-26.75] | 25.92 [12.59-49.65] | 82.28 | 8.3 [3.89-15.72] | 7.7 [3.72-14.33] | -0.14 [-0.22 to -0.06] |
| Central African  Republic | 1.08 [0.58-1.95] | 1.79 [0.93-3.17] | 65.74 | 15.03 [7.9-26.7] | 12.85 [6.69-23.19] | -0.51 [-0.57 to -0.45] |
| Chad | 3.25 [1.68-6.05] | 5.77 [2.86-10.78] | 77.54 | 19.51 [9.74-36.25] | 17.29 [8.37-32.63] | -0.44 [-0.46 to -0.42] |
| Chile | 6.27 [3.04-11.97] | 11.34 [5.3-21.31] | 80.86 | 11.1 [5.42-21.13] | 8.99 [4.23-16.84] | -0.86 [-0.95 to -0.76] |
| China | 268.93 [130.59-511.19] | 532.35 [267.82-994.2] | 97.95 | 6.03 [2.92-11.44] | 5.05 [2.57-9.35] | -0.13 [-0.31 to 0.06] |
| Colombia | 14.69 [7.37-26.89] | 31.29 [15.16-59.4] | 113.00 | 14.78 [7.39-27.16] | 10.92 [5.29-20.74] | -1.03 [-1.1 to -0.95] |
| Comoros | 0.16 [0.08-0.29] | 0.27 [0.13-0.49] | 68.75 | 12.83 [6.63-23.37] | 9.27 [4.67-16.98] | -1.18 [-1.21 to -1.15] |
| Congo | 0.97 [0.52-1.71] | 1.88 [0.98-3.41] | 93.81 | 14.65 [7.84-26.06] | 11.43 [5.91-20.64] | -0.69 [-0.79 to -0.6] |
| Costa Rica | 1.35 [0.66-2.61] | 2.68 [1.24-5.15] | 98.52 | 14.21 [6.85-27.72] | 9.56 [4.43-18.42] | -1.34 [-1.45 to -1.23] |
| Cote d'Ivoire | 4.82 [2.49-8.42] | 9.56 [4.64-18.08] | 98.34 | 18.25 [9.09-33.22] | 14.63 [7.09-27.88] | NA |
| Croatia | 2.27 [1.05-4.33] | 2.31 [1.1-4.44] | 1.76 | 6.11 [2.81-11.58] | 5.37 [2.56-10.47] | -0.45 [-0.68 to -0.21] |
| Cuba | 7.43 [3.96-13.27] | 10.54 [5.45-19.34] | 41.86 | 14.19 [7.57-25.44] | 11.76 [6.04-21.61] | -0.5 [-0.6 to -0.39] |
| Cyprus | 0.46 [0.22-0.88] | 0.71 [0.34-1.33] | 54.35 | 10.79 [5.18-20.41] | 7.53 [3.63-14.1] | -1.79 [-1.99 to -1.59] |
| Czechia | 6.02 [2.78-11.57] | 7.76 [3.57-14.87] | 28.90 | 8.08 [3.69-15.74] | 7.78 [3.58-14.91] | 0.5 [0.19 to 0.8] |
| Democratic  People's Republic  of Korea | 7.89 [3.8-14.87] | 9.77 [4.75-18.22] | 23.83 | 7.68 [3.72-14.56] | 5.35 [2.63-9.98] | -1.22 [-1.25 to -1.19] |
| Democratic  Republic of the  Congo | 16.24 [8.41-29.07] | 29.74 [16.01-52.71] | 83.13 | 16.36 [8.4-29.34] | 12.98 [6.83-22.83] | -0.74 [-0.86 to -0.62] |
| Denmark | 4.47 [2.17-8.49] | 5.68 [2.71-10.86] | 27.07 | 11.73 [5.59-22.38] | 11.77 [5.5-22.88] | 0.05 [0 to 0.1] |
| Djibouti | 0.13 [0.07-0.23] | 0.38 [0.19-0.69] | 192.31 | 13.33 [7.18-24.17] | 10.08 [5.03-18.49] | -0.99 [-1.07 to -0.91] |
| Dominica | 0.06 [0.03-0.11] | 0.05 [0.03-0.09] | −16.67 | 17.99 [9.72-31.28] | 13 [6.98-22.84] | -1.18 [-1.23 to -1.13] |
| Dominican  Republic | 4.32 [2.34-7.67] | 7.42 [3.88-13.57] | 71.76 | 18.03 [9.32-33.11] | 14.43 [7.44-26.78] | -0.86 [-0.94 to -0.78] |
| Ecuador | 6.98 [3.91-12.27] | 11.88 [6.05-21.93] | 70.20 | 21.01 [11.14-37.17] | 14.44 [7.39-26.52] | -1.22 [-1.44 to -1.01] |
| Egypt | 34.36 [19.4-58.98] | 59.48 [31.33-106.24] | 73.11 | 17.72 [9.54-31.22] | 15.26 [7.87-27.21] | -0.36 [-0.43 to -0.3] |
| El Salvador | 2.7 [1.41-4.89] | 3.81 [1.96-6.97] | 41.11 | 15.66 [7.88-28.62] | 11.22 [5.75-20.6] | -1.15 [-1.28 to -1.02] |
|  |  |  |  |  |  |  |
| Equatorial Guinea | 0.21 [0.1-0.37] | 0.39 [0.2-0.69] | 85.71 | 16.68 [8.29-30.16] | 11.11 [5.8-19.92] | -1.48 [-1.57 to -1.39] |
| Eritrea | 0.9 [0.47-1.63] | 1.92 [0.95-3.5] | 113.33 | 12.53 [6.41-22.92] | 10.14 [5.11-18.89] | -0.79 [-0.84 to -0.73] |
| Estonia | 0.95 [0.44-1.85] | 0.98 [0.46-1.87] | 3.16 | 7.66 [3.52-14.85] | 7.22 [3.35-13.76] | 0.54 [0.19 to 0.89] |
| Eswatini | 0.24 [0.12-0.44] | 0.39 [0.19-0.72] | 62.50 | 12.36 [5.99-23.02] | 10.22 [4.86-18.95] | -0.6 [-0.64 to -0.56] |
| Ethiopia | 16.72 [9.11-29.03] | 30.52 [15.62-54.34] | 82.54 | 14.04 [7.59-24.66] | 11.75 [5.99-20.85] | -0.68 [-0.71 to -0.65] |
| Fiji | 0.17 [0.09-0.32] | 0.34 [0.19-0.57] | 100.00 | 7.51 [4.03-13.44] | 7.83 [4.39-13.52] | 0.39 [0.23 to 0.54] |
| Finland | 4.2 [2.05-8.03] | 5.13 [2.39-9.92] | 22.14 | 11.33 [5.41-22] | 10.36 [4.81-19.96] | -0.43 [-0.54 to -0.32] |
| France | 45.55 [23.01-84.62] | 62.96 [30.18-119.44] | 38.22 | 11.16 [5.53-21] | 10.96 [5.21-21.09] | 0.13 [0.05 to 0.2] |
| Gabon | 0.47 [0.25-0.84] | 0.68 [0.35-1.23] | 44.68 | 14.56 [7.7-26] | 10.52 [5.51-19.02] | -1.14 [-1.17 to -1.11] |
| Gambia | 0.41 [0.21-0.72] | 0.8 [0.4-1.5] | 95.12 | 19 [9.47-35.18] | 13.89 [6.63-26.68] | -1.14 [-1.19 to -1.08] |
| Germany | 73.89 [36.26-138.33] | 88.81 [42.83-174.83] | 20.19 | 11.51 [5.69-21.65] | 11.36 [5.42-21.61] | 0.19 [0.08 to 0.3] |
| Ghana | 7.11 [3.69-12.9] | 14.02 [6.66-27.07] | 97.19 | 17.45 [8.95-32.37] | 13.09 [6.17-25.24] | -0.97 [-1.21 to -0.73] |
| Greece | 6.52 [3.08-12.44] | 8.66 [4.1-16.43] | 32.82 | 8.8 [4.16-16.63] | 8.96 [4.22-16.98] | 0.34 [0.21 to 0.47] |
| Greenland | 0.02 [0.01-0.03] | 0.03 [0.01-0.05] | 50.00 | 9.12 [4.26-17.07] | 8.28 [4.05-15.21] | -0.4 [-0.51 to -0.29] |
| Grenada | 0.07 [0.04-0.12] | 0.08 [0.04-0.14] | 14.29 | 19.68 [11.32-34.12] | 13.24 [7.3-23.77] | -1.23 [-1.43 to -1.04] |
| Guam | 0.03 [0.02-0.06] | 0.06 [0.03-0.12] | 100.00 | 7.37 [3.95-13.29] | 6.59 [3.28-12.25] | -0.51 [-0.63 to -0.38] |
| Guatemala | 4.51 [2.46-8.02] | 7.07 [3.4-13.57] | 56.76 | 19.98 [10.77-36.01] | 10.9 [5.15-20.83] | -2.18 [-2.45 to -1.92] |
| Guinea | 3.55 [1.81-6.56] | 4.84 [2.32-8.93] | 36.34 | 18.93 [9.43-35.21] | 14.79 [7.05-28] | -0.9 [-0.93 to -0.87] |
| Guinea-Bissau | 0.47 [0.24-0.87] | 0.7 [0.34-1.33] | 48.94 | 18.16 [8.96-33.95] | 14.3 [6.72-27.21] | -0.86 [-0.91 to -0.81] |
| Guyana | 0.4 [0.21-0.71] | 0.44 [0.23-0.82] | 10.00 | 17.09 [9.45-30.22] | 12.11 [6.17-22.3] | -1.15 [-1.2 to -1.11] |
| Haiti | 4.03 [2.12-7.34] | 6.68 [3.41-12.19] | 65.76 | 20.28 [10.57-36.73] | 14.35 [7.33-26.09] | -1.15 [-1.19 to -1.11] |
| Honduras | 2.21 [1.17-3.96] | 4.24 [2.15-7.85] | 91.86 | 19.04 [9.9-34.23] | 12.21 [6.23-22.39] | -1.59 [-1.71 to -1.47] |
| Hungary | 6.81 [3.34-12.95] | 6.95 [3.36-13.17] | 2.06 | 8.32 [4.04-15.75] | 7.08 [3.33-13.44] | -0.34 [-0.55 to -0.12] |
| Iceland | 0.2 [0.1-0.38] | 0.32 [0.15-0.61] | 60.00 | 14.49 [6.9-27.68] | 12.92 [6.01-24.92] | -1.42 [-1.46 to -1.37] |
| India | 767.82 [461.6-1271.15] | 1137.57 [682.13-1927.4] | 48.16 | 26.94 [15.96-44.9] | 17.68 [10.63-30.1] | -1.42 [-1.46 to -1.37] |
| Indonesia | 47.77 [27.32-86.9] | 88.45 [51.9-154.17] | 85.16 | 7.62 [4.22-13.58] | 6.91 [4.03-12.02] | -0.31 [-0.36 to -0.26] |
| Iran (Islamic  Republic of) | 26.21 [12.68-49.8] | 62.48 [29.97-119.93] | 138.38 | 17.43 [8.39-32.79] | 14.49 [7.02-27.46] | -0.41 [-0.6 to -0.23] |
| Iraq | 7.32 [3.6-13.98] | 17.89 [8.44-35.36] | 144.40 | 15.99 [7.66-30.48] | 12.27 [5.74-23.77] | -0.98 [-1.07 to -0.9] |
| Ireland | 2.25 [1.09-4.25] | 3.71 [1.76-7.11] | 64.89 | 11.33 [5.52-21.54] | 10.58 [5.01-20.42] | -0.01 [-0.13 to 0.1] |
| Israel | 3.11 [1.48-5.98] | 7.2 [3.35-13.93] | 131.51 | 12.62 [6.02-24.43] | 13.09 [6.08-25.64] | 0.22 [0.17 to 0.27] |
| Italy | 35.45 [17.33-66.45] | 42.72 [20.98-78.9] | 20.51 | 8.08 [3.92-15.31] | 7.16 [3.53-13.42] | -0.63 [-0.98 to -0.27] |
| Jamaica | 1.43 [0.74-2.61] | 1.96 [1.12-3.41] | 37.06 | 16.02 [8.18-29.29] | 12.76 [7.3-22.18] | -0.74 [-0.78 to -0.69] |
| Japan | 35.57 [17.36-67.25] | 54.69 [26.08-100.96] | 53.75 | 3.76 [1.84-7.11] | 3.25 [1.58-6.18] | -0.47 [-0.57 to -0.37] |
| Jordan | 1.05 [0.51-2.04] | 3.96 [1.84-7.9] | 277.14 | 12.33 [5.87-23.57] | 9.73 [4.57-19.13] | -0.63 [-0.75 to -0.51] |
| Kazakhstan | 4.79 [2.26-9.02] | 6.2 [3-11.57] | 29.44 | 6.12 [2.9-11.48] | 5.89 [2.89-10.98] | 0.35 [0.04 to 0.65] |
| Kenya | 6.79 [3.61-11.99] | 14.43 [7.39-25.54] | 112.52 | 13.27 [7.05-23.96] | 9.89 [5.03-17.83] | -0.96 [-1.02 to -0.89] |
| Kuwait | 0.36 [0.17-0.73] | 1.58 [0.73-3.21] | 338.89 | 10.11 [4.8-19.98] | 8.17 [3.9-15.92] | -1.23 [-1.67 to -0.8] |
| Kyrgyzstan | 1.25 [0.62-2.33] | 1.63 [0.77-3.07] | 30.40 | 6.96 [3.44-13.01] | 5.92 [2.84-10.95] | -0.09 [-0.38 to 0.2] |
| Lao People's  Democratic  Republic | 0.97 [0.49-1.86] | 1.79 [0.91-3.23] | 84.54 | 8.08 [4.03-15.48] | 6.83 [3.39-12.53] | -0.73 [-0.79 to -0.67] |
| Latvia | 1.57 [0.74-2.95] | 1.48 [0.7-2.86] | −5.73 | 7.2 [3.38-13.56] | 7 [3.3-13.4] | 0.18 [-0.23 to 0.59] |
| Lebanon | 1.82 [0.86-3.48] | 3.3 [1.54-6.29] | 81.32 | 14.26 [6.73-27.24] | 11.55 [5.33-21.98] | -0.71 [-0.73 to -0.69] |
| Lesotho | 0.68 [0.33-1.3] | 0.77 [0.38-1.45] | 13.24 | 11.86 [5.73-22.48] | 9.72 [4.67-18.32] | -0.62 [-0.69 to -0.55] |
| Liberia | 1.07 [0.55-1.96] | 1.74 [0.83-3.26] | 62.62 | 18.44 [9.14-34.17] | 13.56 [6.36-25.57] | -1.1 [-1.14 to -1.05] |
| Libya | 1.37 [0.65-2.67] | 3.19 [1.49-6.48] | 132.85 | 13.91 [6.58-26.84] | 9.8 [4.58-18.9] | -0.97 [-1.1 to -0.84] |
| Lithuania | 2.18 [1.02-4.13] | 2.35 [1.12-4.49] | 7.80 | 8.09 [3.74-15.47] | 7.68 [3.55-14.34] | 0.04 [-0.18 to 0.25] |
| Luxembourg | 0.27 [0.13-0.51] | 0.43 [0.2-0.8] | 59.26 | 9.66 [4.55-18.43] | 9.19 [4.34-17.45] | -0.74 [-0.91 to -0.58] |
| Madagascar | 3.82 [2.01-7.09] | 7.63 [3.81-14.02] | 99.74 | 12.62 [6.61-23.4] | 10.38 [5.19-19.64] | -0.68 [-0.73 to -0.64] |
| Malawi | 3.29 [1.77-6.05] | 5.39 [2.76-9.75] | 63.83 | 13.53 [7.22-24.87] | 11.18 [5.63-20.48] | -0.68 [-0.75 to -0.62] |
| Malaysia | 3.37 [1.68-6.34] | 7.89 [3.92-14.75] | 134.12 | 6.42 [3.13-12.01] | 5.55 [2.79-10.3] | -0.64 [-0.72 to -0.57] |
| Maldives | 0.06 [0.03-0.12] | 0.14 [0.08-0.22] | 133.33 | 10.47 [5.12-19.61] | 7.82 [4.61-12.75] | -0.72 [-0.87 to -0.57] |
| Mali | 4.93 [2.5-9.03] | 8.59 [4.18-16.01] | 74.24 | 19.9 [10.13-37.3] | 16.87 [8.12-31.45] | -0.64 [-0.67 to -0.61] |
| Malta | 0.23 [0.11-0.44] | 0.35 [0.17-0.67] | 52.17 | 10.03 [4.78-19.09] | 9.03 [4.3-17.47] | -0.51 [-0.89 to -0.13] |
| Marshall Islands | 0.01 [0-0.01] | 0.01 [0.01-0.03] | 0.00 | 7.88 [4.08-14.48] | 6.89 [3.54-12.49] | -0.34 [-0.4 to -0.28] |
| Mauritania | 1.07 [0.54-1.95] | 1.63 [0.78-3.1] | 52.34 | 18.06 [8.94-33.44] | 13.48 [6.42-25.64] | -0.98 [-1.03 to -0.93] |
| Mauritius | 0.23 [0.11-0.42] | 0.42 [0.2-0.8] | 82.61 | 5.46 [2.65-10.17] | 4.42 [2.07-8.37] | -0.81 [-0.85 to -0.76] |
| Mexico | 37.4 [20.71-66.23] | 81.06 [40.78-152.68] | 116.74 | 15.35 [8.51-27.32] | 12.46 [6.26-23.33] | -1.15 [-1.34 to -0.96] |
| Micronesia | 0.02 [0.01-0.04] | 0.03 [0.01-0.05] | 50.00 | 8.2 [4.34-14.8] | 6.43 [3.26-11.58] | -0.78 [-0.83 to -0.72] |
| Mongolia | 1.26 [0.35-2.49] | 1.73 [0.74-3.19] | 37.30 | 16.44 [5.79-31.39] | 10.29 [4.94-17.92] | -1.66 [-2 to -1.32] |
| Montenegro | 0.32 [0.17-0.6] | 0.38 [0.18-0.71] | 18.75 | 9.23 [4.86-17.08] | 7.49 [3.66-13.85] | -0.75 [-0.85 to -0.65] |
| Morocco | 10.78 [5.08-20.64] | 21.25 [9.8-41.15] | 97.12 | 13.54 [6.26-25.79] | 11.8 [5.47-22.75] | -0.46 [-0.52 to -0.39] |
| Mozambique | 4.64 [2.36-8.5] | 8.39 [4.19-15.42] | 80.82 | 12.29 [6.28-22.84] | 11.28 [5.64-20.96] | -0.3 [-0.34 to -0.25] |
| Myanmar | 9.6 [4.77-18.37] | 17.83 [9.35-32.44] | 85.73 | 7.16 [3.5-13.74] | 6.36 [3.34-11.67] | -0.34 [-0.39 to -0.29] |
| Namibia | 0.49 [0.24-0.93] | 0.95 [0.46-1.74] | 93.88 | 12.02 [5.76-22.47] | 10.72 [5.19-19.84] | -0.31 [-0.36 to -0.27] |
| Nepal | 24.81 [13.47-42.94] | 31.56 [17.48-53.44] | 27.21 | 41.15 [22.16-71.36] | 23.09 [12.94-39.56] | -2.06 [-2.28 to -1.84] |
| Netherlands | 11.14 [5.81-20.48] | 15.44 [7.68-28.81] | 38.60 | 11.05 [5.68-20.31] | 10.54 [5.15-19.61] | 0.08 [-0.01 to 0.18] |
| New Zealand | 2.12 [1.09-3.84] | 3.74 [1.77-6.98] | 76.42 | 10.54 [5.42-19.23] | 9.9 [4.67-18.62] | -0.46 [-1.03 to 0.13] |
| Nicaragua | 1.61 [0.82-2.94] | 2.99 [1.45-5.59] | 85.71 | 17.12 [8.27-32.23] | 11.22 [5.49-21.03] | -1.41 [-1.52 to -1.3] |
| Niger | 3.61 [1.86-6.62] | 9.27 [4.54-17.24] | 156.79 | 20.38 [10.17-38.19] | 18.59 [8.96-35.32] | -0.37 [-0.4 to -0.35] |
| Nigeria | 44.76 [23.7-80.03] | 87.19 [43.32-161.71] | 94.79 | 19.08 [9.75-34.8] | 14.69 [7.31-27.18] | -0.93 [-0.97 to -0.88] |
| North Macedonia | 0.89 [0.45-1.68] | 1.09 [0.52-2.08] | 22.47 | 8.62 [4.32-16.14] | 6.71 [3.26-12.7] | -0.85 [-1.02 to -0.68] |
| Northern Mariana  Islands | 0.01 [0-0.01] | 0.02 [0.01-0.03] | 100.00 | 7.09 [3.9-12.43] | 6.15 [3.3-10.91] | -0.41 [-0.47 to -0.35] |
| Norway | 4.22 [2.14-7.69] | 5.4 [2.64-9.92] | 27.96 | 13.78 [6.85-25.47] | 13.15 [6.41-24.18] | -0.47 [-0.57 to -0.36] |
| Oman | 0.6 [0.28-1.21] | 1.17 [0.53-2.33] | 95.00 | 16.26 [7.61-31.87] | 10.63 [4.91-20.36] | -1.63 [-1.69 to -1.57] |
| Pakistan | 132.74 [71.16-228.88] | 200.96 [122.09-329.6] | 51.39 | 41.22 [22.01-73.31] | 26.47 [15.69-45.07] | -1.55 [-1.62 to -1.48] |
| Palestine | 0.87 [0.42-1.67] | 1.99 [0.92-3.93] | 128.74 | 16.5 [7.83-31.53] | 12.9 [6.06-25.07] | -0.93 [-1.09 to -0.78] |
| Panama | 1.2 [0.64-2.17] | 2.29 [1.07-4.41] | 90.83 | 15.15 [7.96-27.5] | 10.73 [5.05-20.74] | -1.08 [-1.25 to -0.92] |
| Papua New Guinea | 0.78 [0.4-1.41] | 2.09 [1.08-3.81] | 167.95 | 7.52 [3.78-13.75] | 7.51 [3.86-13.77] | 0.01 [0 to 0.01] |
| Paraguay | 2.77 [1.42-5.1] | 4.66 [2.25-8.71] | 68.23 | 19.81 [10.07-36.86] | 14.46 [6.94-26.99] | -0.99 [-1.04 to -0.93] |
| Peru | 14.69 [8.21-25.95] | 25.16 [12.73-46.02] | 71.27 | 20.71 [11.25-37.1] | 14.64 [7.38-26.72] | -1.25 [-1.34 to -1.15] |
| Philippines | 13.65 [7.38-24.58] | 32.88 [18.5-56.44] | 140.88 | 7.6 [4.07-13.52] | 7.15 [4-12.38] | -0.16 [-0.19 to -0.14] |
| Poland | 22.4 [11.16-41.69] | 34.15 [16.49-64.02] | 52.46 | 9.16 [4.55-16.97] | 9.66 [4.64-18.29] | 0.53 [-0.17 to 1.23] |
| Portugal | 5.4 [2.61-10.1] | 6.24 [2.94-11.96] | 15.56 | 7.6 [3.66-14.45] | 6.06 [2.93-11.93] | -0.82 [-0.99 to -0.66] |
| Puerto Rico | 2.81 [1.42-5.17] | 2.89 [1.39-5.41] | 2.85 | 14.71 [7.42-27.01] | 9.56 [4.6-18.12] | -1.48 [-1.58 to -1.38] |
| Qatar | 0.09 [0.04-0.18] | 0.55 [0.25-1.14] | 511.11 | 13.2 [6.29-25.75] | 9.83 [4.58-19.27] | -1.05 [-1.09 to -1.01] |
| Republic of Korea | 9.22 [5.15-16.03] | 18.37 [9.03-34.24] | 99.24 | 5.07 [2.77-8.97] | 3.93 [1.95-7.19] | -1.06 [-1.28 to -0.84] |
| Republic of  Moldova | 2.21 [1.04-4.21] | 2.1 [0.97-3.97] | −4.98 | 8.4 [3.97-15.88] | 6.41 [2.98-12.06] | -0.49 [-0.81 to -0.17] |
| Romania | 10.59 [5.34-19.67] | 11.48 [5.3-21.94] | 8.40 | 6.8 [3.45-12.64] | 6.19 [2.81-11.91] | -0.04 [-0.34 to 0.26] |
| Russian Federation | 86.24 [43.94-160.97] | 103.56 [51.95-195.01] | 20.08 | 7.64 [3.91-14.32] | 7.6 [3.81-14.34] | 0.76 [0.3 to 1.23] |
| Rwanda | 2.39 [1.22-4.33] | 4.26 [2.16-7.91] | 78.24 | 12.9 [6.58-23.81] | 10.27 [5.11-19.18] | -0.86 [-0.89 to -0.83] |
| Saint Lucia | 0.07 [0.03-0.14] | 0.11 [0.05-0.2] | 57.14 | 14.62 [6.85-27.92] | 9.54 [4.49-18.17] | -1.45 [-1.53 to -1.37] |
| Saint Vincent and  the Grenadines | 0.06 [0.03-0.11] | 0.08 [0.04-0.15] | 33.33 | 15.51 [7.44-29.49] | 11.81 [5.83-22.21] | -1.02 [-1.09 to -0.95] |
| Samoa | 0.03 [0.02-0.05] | 0.04 [0.02-0.07] | 33.33 | 6.17 [3.26-11.26] | 4.85 [2.52-8.76] | -0.83 [-0.88 to -0.77] |
| Sao Tome and  Principe | 0.07 [0.04-0.12] | 0.1 [0.05-0.18] | 42.86 | 18.57 [9.22-34.69] | 14.23 [7.05-26.23] | -0.94 [-1.04 to -0.84] |
| Saudi Arabia | 4.73 [2.21-9.08] | 12.74 [5.84-24.78] | 169.34 | 14.93 [6.95-28.17] | 10.24 [4.74-19.86] | -1.41 [-1.49 to -1.32] |
| Senegal | 3.63 [1.89-6.6] | 6.56 [3.22-12.3] | 80.72 | 18.52 [9.25-34.22] | 14.44 [6.99-27.11] | -0.81 [-0.86 to -0.75] |
| Serbia | 4.58 [2.19-8.52] | 4.64 [2.31-8.47] | 1.31 | 7.26 [3.58-13.24] | 6.06 [3.1-11.14] | -0.69 [-0.89 to -0.49] |
| Seychelles | 0.02 [0.01-0.04] | 0.04 [0.02-0.07] | 100.00 | 7.04 [3.76-12.65] | 6.59 [3.49-11.71] | -0.14 [-0.24 to -0.04] |
| Sierra Leone | 1.86 [0.94-3.45] | 3.1 [1.51-5.85] | 66.67 | 17.55 [8.73-33.24] | 14.43 [6.89-27.97] | -0.7 [-0.75 to -0.66] |
| Singapore | 0.53 [0.26-0.98] | 1.47 [0.71-2.82] | 177.36 | 4.41 [2.17-8.1] | 3.63 [1.75-6.97] | -0.67 [-0.84 to -0.51] |
| Slovakia | 3.36 [1.79-6.03] | 3.85 [1.83-7.47] | 14.58 | 10.47 [5.63-18.84] | 8.11 [3.85-15.52] | -0.64 [-0.97 to -0.3] |
| Slovenia | 1.2 [0.58-2.25] | 1.68 [0.77-3.31] | 40.00 | 8.58 [4.08-16.12] | 8.32 [3.69-16.27] | 0.26 [0.06 to 0.45] |
| Solomon Islands | 0.07 [0.04-0.13] | 0.16 [0.08-0.29] | 128.57 | 9.23 [4.74-16.74] | 8.27 [4.31-14.9] | -0.38 [-0.41 to -0.35] |
| Somalia | 2.37 [1.23-4.4] | 5.73 [2.91-10.34] | 141.77 | 13.58 [7.03-25.19] | 12.35 [6.3-22.63] | -0.32 [-0.36 to -0.28] |
| South Africa | 14.27 [7.52-25.94] | 25.76 [13.43-47.26] | 80.52 | 11.2 [5.9-20.35] | 9.52 [4.99-17.37] | -0.58 [-0.62 to -0.55] |
| South Sudan | 1.51 [0.79-2.74] | 2.75 [1.42-4.98] | 82.12 | 12.1 [6.17-22.16] | 11.21 [5.75-20.58] | -0.28 [-0.31 to -0.24] |
| Spain | 24.81 [11.92-46.72] | 37.83 [18.14-72.62] | 52.48 | 9.24 [4.45-17.62] | 9.12 [4.34-17.59] | 0.22 [0.12 to 0.33] |
| Sri Lanka | 4.63 [2.63-8.33] | 8.26 [4.14-15.43] | 78.40 | 7.2 [3.99-12.89] | 5.96 [3-11.2] | -0.65 [-0.68 to -0.62] |
| Sudan | 8.48 [3.93-16.59] | 15.18 [7.18-29.39] | 79.01 | 16.13 [7.5-30.9] | 12.96 [6.11-24.77] | -0.77 [-0.87 to -0.67] |
| Suriname | 0.23 [0.11-0.42] | 0.39 [0.2-0.74] | 69.57 | 15.25 [7.6-28.36] | 12.02 [6.02-22.48] | -0.77 [-0.85 to -0.69] |
| Sweden | 6.58 [3.24-12.18] | 6.72 [3.29-12.41] | 2.13 | 10.17 [4.96-18.84] | 7.75 [3.82-14.53] | -0.89 [-1.04 to -0.73] |
| Switzerland | 5.34 [2.52-10.11] | 7.95 [3.86-14.69] | 48.88 | 10.63 [5.05-20.19] | 10.65 [5.13-20.09] | -0.07 [-0.3 to 0.17] |
| Syrian Arab  Republic | 9.85 [5.02-17.34] | 11.56 [6.41-20.15] | 17.36 | 22.17 [12.09-39.01] | 14.89 [8.33-25.96] | -1.07 [-1.28 to -0.86] |
| Tajikistan | 1.96 [1.05-3.32] | 3.27 [1.81-5.31] | 66.84 | 12.13 [6.52-20.59] | 9.84 [5.64-16.22] | -0.47 [-0.77 to -0.17] |
| Thailand | 13.22 [6.94-24.19] | 25.83 [12.78-48.56] | 95.39 | 6.15 [3.21-11.53] | 4.72 [2.38-8.86] | -1.01 [-1.07 to -0.95] |
| Timor-Leste | 0.15 [0.07-0.29] | 0.33 [0.18-0.6] | 120.00 | 8.62 [4.21-16.11] | 7.59 [4.03-13.63] | -0.46 [-0.5 to -0.41] |
| Togo | 1.61 [0.83-2.93] | 3.53 [1.71-6.73] | 119.25 | 18.37 [9.31-34.35] | 13.99 [6.71-26.52] | -0.88 [-0.92 to -0.84] |
| Tonga | 0.02 [0.01-0.04] | 0.03 [0.02-0.05] | 50.00 | 7.07 [3.76-13.32] | 6.69 [3.58-12.15] | -0.12 [-0.16 to -0.08] |
| Trinidad and  Tobago | 0.7 [0.37-1.23] | 0.98 [0.51-1.81] | 40.00 | 14.94 [8-26.48] | 10.69 [5.5-19.61] | -0.95 [-1.12 to -0.79] |
| Tunisia | 3.61 [1.71-7.05] | 7.26 [3.43-14.18] | 101.11 | 13.16 [6.18-25.34] | 10.61 [4.98-20.62] | -0.69 [-0.72 to -0.66] |
| Turkey | 26.57 [13.08-50.44] | 51.61 [25.54-97.26] | 94.24 | 12.64 [6.19-23.96] | 10.79 [5.34-20.19] | -0.46 [-0.51 to -0.41] |
| Turkmenistan | 0.85 [0.42-1.57] | 1.45 [0.7-2.65] | 70.59 | 7.6 [3.7-13.99] | 6.21 [3.02-11.21] | -0.43 [-0.72 to -0.14] |
| Uganda | 5.33 [2.83-9.68] | 11.67 [5.99-21] | 118.95 | 13.59 [7.2-24.92] | 11.82 [5.86-22.19] | -0.38 [-0.55 to -0.21] |
| Ukraine | 34.15 [16.72-64.3] | 29.83 [15-55.65] | −12.65 | 7.81 [3.79-14.73] | 6.79 [3.36-12.78] | 0.19 [-0.24 to 0.62] |
| United Arab  Emirates | 0.33 [0.15-0.67] | 2.17 [0.96-4.33] | 557.58 | 12.93 [6.07-25.07] | 9.02 [4.25-17.88] | -1.1 [-1.18 to -1.03] |
| United Kingdom | 49.78 [25.31-92.35] | 60.49 [29.95-110.76] | 21.51 | 11.58 [5.79-21.76] | 11.46 [5.58-21.31] | 0.17 [0.04 to 0.3] |
| United Republic of  Tanzania | 8.3 [4.31-15.57] | 17.55 [8.7-31.54] | 111.45 | 12.62 [6.51-23.73] | 11.12 [5.51-20.37] | -0.4 [-0.45 to -0.36] |
| United States of  America | 148.52 [73.14-277.87] | 216.13 [106.07-395.03] | 45.52 | 8.97 [4.44-16.97] | 7.64 [3.71-14.06] | -0.91 [-1.05 to -0.78] |
| United States Virgin  Islands | 0.08 [0.04-0.16] | 0.1 [0.05-0.18] | 25.00 | 15.71 [7.7-29.45] | 11.41 [5.52-21.83] | -1.15 [-1.18 to -1.11] |
| Uruguay | 2.22 [1.06-4.28] | 2.47 [1.17-4.74] | 11.26 | 11.08 [5.28-21.42] | 9.48 [4.44-18.2] | -0.51 [-0.62 to -0.4] |
| Uzbekistan | 4.78 [2.19-9.04] | 7.69 [3.46-14.71] | 60.88 | 7.31 [3.33-13.8] | 5.79 [2.67-11.09] | -0.55 [-0.83 to -0.27] |
| Vanuatu | 0.03 [0.01-0.05] | 0.07 [0.03-0.12] | 133.33 | 7.93 [4-14.69] | 7 [3.59-12.85] | -0.48 [-0.52 to -0.45] |
| Venezuela  (Bolivarian Republic  of) | 8.32 [4.01-15.58] | 16.68 [7.85-31.2] | 100.48 | 15.08 [7.28-28.54] | 10.49 [4.94-19.5] | -1.04 [-1.15 to -0.93] |
| Viet Nam | 14.54 [6.85-27.71] | 27.37 [12.53-52.43] | 88.24 | 6.22 [2.92-11.81] | 4.96 [2.28-9.45] | -0.7 [-0.75 to -0.64] |
| Yemen | 5.06 [2.39-9.67] | 12.19 [5.66-23.42] | 140.91 | 16.86 [7.79-31.71] | 13.94 [6.5-26.55] | -0.68 [-0.8 to -0.56] |
| Zambia | 2.38 [1.26-4.27] | 5.07 [2.62-9.09] | 113.03 | 13.4 [6.97-24.17] | 11.2 [5.65-20.32] | -0.62 [-0.65 to -0.58] |
| Zimbabwe | 2.99 [1.47-5.62] | 5.07 [2.42-9.49] | 69.57 | 12.46 [5.91-23.86] | 11.05 [5.25-20.77] | -0.25 [-0.33 to -0.17] |
