# Supplementary material for: Global burden and trends of pelvic organ prolapse associated with aging women: An observational trend study from 1990 to 2019
Source: Front Public Health. 2022 Sep 15;10:975829. doi: 10.3389/fpubh.2022.975829 (PMC9521163; doi:10.3389/fpubh.2022.975829)
Supplement: Supplementary Table 3 — The death and age standardized death rate of POP and the trend of time changes from 1990 to 2019. [file Data_Sheet_3.docx]

Supplementary Table3. The death and age standardized death rate of POP and the trend of time changes from 1990 to 2019

| Nation | Death cases No.(95% UI) | | Rate of change (%) | ASDR per 100,000 No.(95% UI) | | 1990-2019  EAPC No. (95% CI) |
| --- | --- | --- | --- | --- | --- | --- |
| Afghanistan | 0 [0-0] | 0 [0-0.01] | NaN | 0 [0-0.01] | 0 [0-0.01] | 1.02 [0.73 to 1.32] |
| Albania | 0.01 [0.01-0.03] | 0.02 [0.01-0.04] | 100.00 | 0.1 [0.06-0.27] | 0.1 [0.06-0.21] | -0.79 [-1.39 to -0.17] |
| Algeria | 0 [0-0.01] | 0 [0-0.01] | NaN | 0 [0-0.01] | 0 [0-0] | 1.43 [0.81 to 2.05] |
| American Samoa | 0 [0-0] | 0 [0-0] | NaN | 0.11 [0.05-0.21] | 0.07 [0.03-0.12] | -1.23 [-1.77 to -0.68] |
| Andorra | 0 [0-0] | 0 [0-0] | NaN | 0.02 [0-0.03] | 0.01 [0-0.02] | -0.91 [-0.97 to -0.86] |
| Angola | 0.03 [0-0.07] | 0.06 [0.01-0.13] | 100.00 | 0.15 [0.02-0.44] | 0.1 [0.01-0.22] | -1.56 [-1.81 to -1.32] |
| Antigua and  Barbuda | 0 [0-0] | 0 [0-0] | NaN | 0 [0-0] | 0 [0-0] | -1.88 [-2.61 to -1.13] |
| Argentina | 0.04 [0.01-0.05] | 0.01 [0.01-0.03] | −75.00 | 0.02 [0.01-0.03] | 0 [0-0.01] | -6.56 [-7.18 to -5.93] |
| Armenia | 0 [0-0.01] | 0.01 [0.01-0.01] | Inf | 0.03 [0.02-0.06] | 0.03 [0.02-0.05] | 0.2 [-0.15 to 0.55] |
| Australia | 0.03 [0.02-0.04] | 0.02 [0.01-0.04] | −33.33 | 0.03 [0.01-0.04] | 0.01 [0-0.01] | -5.72 [-6.42 to -5.01] |
| Austria | 0.01 [0-0.01] | 0 [0-0] | −100.00 | 0.01 [0-0.01] | 0 [0-0] | -8.16 [-11.11 to -5.12] |
| Azerbaijan | 0.04 [0.01-0.08] | 0.05 [0.01-0.11] | 25.00 | 0.14 [0.03-0.3] | 0.1 [0.03-0.21] | -0.97 [-1.15 to -0.8] |
| Bahamas | 0 [0-0] | 0 [0-0] | NaN | 0.01 [0.01-0.02] | 0 [0-0.01] | -3.72 [-4.55 to -2.88] |
| Bahrain | 0 [0-0] | 0 [0-0] | NaN | 0 [0-0] | 0 [0-0] | 0.98 [0.4 to 1.56] |
| Bangladesh | 0.8 [0.28-1.61] | 1.42 [0.55-3.02] | 77.50 | 0.32 [0.11-0.67] | 0.22 [0.08-0.45] | -1.1 [-1.27 to -0.93] |
| Barbados | 0 [0-0] | 0 [0-0] | NaN | 0.06 [0.04-0.08] | 0.04 [0.02-0.06] | -1.79 [-2.15 to -1.42] |
| Belarus | 0.01 [0-0.01] | 0 [0-0.01] | −100.00 | 0.01 [0-0.02] | 0 [0-0.01] | -4.24 [-4.41 to -4.08] |
| Belgium | 0 [0-0] | 0 [0-0.01] | NaN | 0 [0-0] | 0 [0-0] | -3.44 [-7.28 to 0.56] |
| Belize | 0 [0-0] | 0 [0-0] | NaN | 0.07 [0.05-0.1] | 0.04 [0.03-0.06] | -1.97 [-2.58 to -1.36] |
| Benin | 0.01 [0-0.01] | 0.01 [0-0.01] | 0.00 | 0.04 [0-0.09] | 0.02 [0-0.04] | -3.4 [-3.58 to -3.23] |
|  |  |  |  |  |  |  |
|  |  |  |  |  |  |  |
| Bermuda | 0 [0-0] | 0 [0-0] | NaN | 0.08 [0.05-0.1] | 0.04 [0.02-0.05] | -3.09 [-3.43 to -2.74] |
| Bhutan | 0 [0-0.01] | 0.01 [0-0.02] | Inf | 0.35 [0.1-0.86] | 0.29 [0.11-0.58] | -0.45 [-0.57 to -0.34] |
| Bolivia  (Plurinational State  of) | 0.02 [0.01-0.03] | 0.03 [0.01-0.06] | 50.00 | 0.1 [0.03-0.21] | 0.07 [0.03-0.13] | -1.41 [-1.65 to -1.17] |
| Bosnia and  Herzegovina | 0.01 [0-0.01] | 0 [0-0.01] | −100.00 | 0.03 [0.01-0.04] | 0.02 [0.01-0.03] | -1.17 [-1.42 to -0.92] |
| Botswana | 0 [0-0] | 0 [0-0] | NaN | 0.03 [0.01-0.07] | 0.02 [0.01-0.06] | -1.42 [-1.51 to -1.33] |
| Brazil | 0.14 [0.11-0.22] | 0.32 [0.24-0.46] | 128.57 | 0.03 [0.03-0.06] | 0.02 [0.02-0.04] | -1.12 [-1.3 to -0.94] |
| Brunei Darussalam | 0 [0-0] | 0 [0-0] | NaN | 0.01 [0.01-0.03] | 0.02 [0.01-0.03] | 0.3 [0.17 to 0.43] |
| Bulgaria | 0 [0-0] | 0 [0-0] | NaN | 0.01 [0-0.01] | 0 [0-0] | -6.16 [-6.78 to -5.53] |
| Burkina Faso | 0.01 [0-0.03] | 0.01 [0-0.02] | 0.00 | 0.05 [0-0.13] | 0.02 [0.01-0.05] | -3.66 [-3.97 to -3.34] |
| Burundi | 0.01 [0-0.02] | 0.01 [0-0.02] | 0.00 | 0.05 [0.01-0.11] | 0.04 [0.01-0.07] | -0.88 [-0.95 to -0.81] |
| Cabo Verde | 0 [0-0] | 0 [0-0] | NaN | 0 [0-0.01] | 0 [0-0] | -0.82 [-1.01 to -0.63] |
| Cambodia | 0.01 [0-0.02] | 0.02 [0.01-0.05] | 100.00 | 0.02 [0.01-0.07] | 0.03 [0.01-0.06] | 1.74 [1.08 to 2.41] |
| Cameroon | 0.01 [0-0.03] | 0.01 [0-0.02] | 0.00 | 0.04 [0-0.08] | 0.01 [0-0.03] | -3.89 [-4.01 to -3.77] |
| Canada | 0.02 [0.01-0.03] | 0.05 [0.02-0.09] | 150.00 | 0.01 [0.01-0.02] | 0.01 [0-0.02] | 0.97 [0.3 to 1.64] |
| Central African  Republic | 0.01 [0-0.02] | 0.01 [0-0.02] | 0.00 | 0.14 [0.02-0.36] | 0.1 [0.01-0.23] | -1.04 [-1.24 to -0.83] |
| Chad | 0.01 [0-0.02] | 0.01 [0-0.02] | 0.00 | 0.05 [0-0.14] | 0.02 [0-0.06] | -3.01 [-3.16 to -2.86] |
| Chile | 0.01 [0-0.01] | 0 [0-0.01] | −100.00 | 0.01 [0.01-0.02] | 0 [0-0.01] | -3.57 [-3.82 to -3.33] |
| China | 0.1 [0.04-0.38] | 0.69 [0.23-0.97] | 590.00 | 0 [0-0.01] | 0.01 [0-0.01] | 6.81 [5.81 to 7.83] |
|  |  |  |  |  |  |  |
|  |  |  |  |  |  |  |
| Colombia | 0.04 [0.03-0.06] | 0.06 [0.04-0.08] | 50.00 | 0.05 [0.03-0.07] | 0.02 [0.01-0.03] | -2.48 [-3.59 to -1.35] |
| Comoros | 0 [0-0] | 0 [0-0] | NaN | 0.04 [0.01-0.09] | 0.03 [0.01-0.06] | -1.92 [-2.17 to -1.68] |
| Congo | 0.01 [0-0.02] | 0.01 [0-0.02] | 0.00 | 0.13 [0.02-0.31] | 0.07 [0.01-0.13] | -1.93 [-2.17 to -1.69] |
| Costa Rica | 0 [0-0] | 0 [0-0] | NaN | 0.01 [0.01-0.02] | 0 [0-0.01] | -4.23 [-6.29 to -2.13] |
| Cote d'Ivoire | 0.01 [0-0.03] | 0.01 [0-0.02] | 0.00 | 0.04 [0-0.1] | 0.01 [0-0.03] | NA |
| Croatia | 0 [0-0] | 0 [0-0] | NaN | 0 [0-0] | 0 [0-0] | 2.12 [-0.52 to 4.82] |
| Cuba | 0.04 [0.02-0.05] | 0.05 [0.03-0.07] | 25.00 | 0.07 [0.05-0.1] | 0.05 [0.03-0.07] | -2.09 [-2.71 to -1.46] |
| Cyprus | 0 [0-0] | 0 [0-0] | NaN | 0 [0-0.01] | 0 [0-0.01] | 0.2 [-0.15 to 0.56] |
| Czechia | 0 [0-0.01] | 0 [0-0] | NaN | 0 [0-0.01] | 0 [0-0] | 7.33 [-8.1 to -6.56] |
| Democratic  People's Republic  of Korea | 0.01 [0-0.02] | 0.01 [0-0.03] | 0.00 | 0.01 [0-0.02] | 0.01 [0-0.02] | 0.03 [-0.26 to 0.32] |
| Democratic  Republic of the  Congo | 0.12 [0.01-0.34] | 0.18 [0.03-0.38] | 50.00 | 0.17 [0.02-0.5] | 0.1 [0.01-0.23] | -1.65 [-1.99 to -1.3] |
| Denmark | 0.01 [0-0.01] | 0 [0-0] | −100.00 | 0.01 [0.01-0.02] | 0 [0-0.01] | -7.69 [-9.6 to -5.74] |
| Djibouti | 0 [0-0] | 0 [0-0] | NaN | 0.05 [0.01-0.11] | 0.03 [0-0.06] | -2.34 [-2.5 to -2.18] |
| Dominica | 0 [0-0] | 0 [0-0] | NaN | 0.08 [0.04-0.17] | 0.06 [0.03-0.1] | -1.3 [-1.49 to -1.12] |
| Dominican  Republic | 0.01 [0-0.02] | 0.02 [0.01-0.03] | 100.00 | 0.03 [0.01-0.05] | 0.03 [0.01-0.06] | -0.97 [-1.52 to -0.42] |
| Ecuador | 0.02 [0.01-0.03] | 0.03 [0.02-0.07] | 50.00 | 0.04 [0.02-0.06] | 0.04 [0.02-0.09] | 0.93 [-0.18 to 2.06] |
| Egypt | 0.15 [0.07-0.26] | 0.23 [0.09-0.49] | 53.33 | 0.06 [0.02-0.1] | 0.05 [0.02-0.1] | -0.34 [-0.54 to -0.13] |
| El Salvador | 0.01 [0-0.01] | 0.01 [0-0.02] | 0.00 | 0.03 [0.01-0.04] | 0.03 [0.01-0.06] | 0.44 [0.28 to 0.6] |
|  |  |  |  |  |  |  |
|  |  |  |  |  |  |  |
| Equatorial Guinea | 0 [0-0] | 0 [0-0] | NaN | 0.18 [0.02-0.53] | 0.06 [0.01-0.12] | -4.02 [-4.2 to -3.84] |
| Eritrea | 0 [0-0.01] | 0 [0-0.01] | NaN | 0.04 [0.01-0.1] | 0.03 [0.01-0.07] | -1.39 [-1.53 to -1.26] |
| Estonia | 0 [0-0] | 0 [0-0] | NaN | 0 [0-0] | 0 [0-0] | -5.42 [-7.09 to -3.72] |
| Eswatini | 0 [0-0] | 0 [0-0] | NaN | 0.03 [0.01-0.09] | 0.02 [0.01-0.06] | -1.81 [-1.97 to -1.64] |
| Ethiopia | 0.07 [0.02-0.16] | 0.09 [0.02-0.19] | 28.57 | 0.07 [0.02-0.14] | 0.03 [0.01-0.07] | -2.61 [-2.72 to -2.5] |
| Fiji | 0 [0-0] | 0 [0-0] | NaN | 0.04 [0.02-0.1] | 0.06 [0.02-0.11] | 2.33 [1.69 to 2.97] |
| Finland | 0 [0-0] | 0 [0-0] | NaN | 0 [0-0.01] | 0 [0-0] | -14.49 [-17.56 to -11.31] |
| France | 0.22 [0.08-0.3] | 0.1 [0.04-0.15] | −54.55 | 0.04 [0.01-0.05] | 0.01 [0-0.01] | -5.17 [-5.61 to -4.74] |
| Gabon | 0 [0-0.01] | 0 [0-0.01] | NaN | 0.12 [0.02-0.31] | 0.06 [0.01-0.11] | -2.66 [-2.76 to -2.57] |
| Gambia | 0 [0-0] | 0 [0-0] | NaN | 0.04 [0-0.1] | 0.02 [0-0.04] | -4.29 [-4.52 to -4.05] |
| Germany | 0.12 [0.06-0.15] | 0.06 [0.04-0.11] | −50.00 | 0.01 [0.01-0.02] | 0.01 [0-0.01] | -2.89 [-3.96 to -1.82] |
| Ghana | 0.02 [0-0.03] | 0.01 [0-0.03] | −50.00 | 0.03 [0-0.07] | 0.01 [0-0.03] | -4.28 [-4.53 to -4.02] |
| Greece | 0 [0-0] | 0 [0-0] | NaN | 0 [0-0] | 0 [0-0] | -3.45 [-4.62 to -2.27] |
| Greenland | 0 [0-0] | 0 [0-0] | NaN | 0.01 [0-0.01] | 0.01 [0-0.02] | -0.88 [-1.4 to -0.35] |
| Grenada | 0 [0-0] | 0 [0-0] | NaN | 0.16 [0.1-0.25] | 0.09 [0.06-0.15] | -1.67 [-2.27 to -1.07] |
| Guam | 0 [0-0] | 0 [0-0] | NaN | 0.03 [0.01-0.05] | 0.01 [0-0.02] | -4.93 [-5.39 to -4.46] |
| Guatemala | 0.02 [0-0.04] | 0.01 [0-0.01] | −50.00 | 0.07 [0.03-0.13] | 0.01 [0.01-0.02] | -6.57 [-8.84 to -4.25] |
| Guinea | 0.01 [0-0.02] | 0.01 [0-0.01] | 0.00 | 0.04 [0-0.11] | 0.02 [0-0.04] | -3.97 [-4.22 to -3.73] |
| Guinea-Bissau | 0 [0-0] | 0 [0-0] | NaN | 0.04 [0-0.09] | 0.01 [0-0.04] | -3.74 [-3.92 to -3.56] |
| Guyana | 0 [0-0] | 0 [0-0] | NaN | 0.12 [0.07-0.18] | 0.05 [0.02-0.08] | -3.48 [-3.91 to -3.04] |
| Haiti | 0.02 [0-0.05] | 0.02 [0.01-0.05] | 0.00 | 0.13 [0.03-0.29] | 0.06 [0.02-0.13] | -2.62 [-2.82 to -2.42] |
|  |  |  |  |  |  |  |
|  |  |  |  |  |  |  |
| Honduras | 0.01 [0-0.02] | 0.02 [0-0.03] | 100.00 | 0.12 [0.02-0.27] | 0.07 [0.01-0.12] | -1.92 [-2.21 to -1.63] |
| Hungary | 0.02 [0-0.03] | 0.02 [0.01-0.02] | 0.00 | 0.02 [0-0.03] | 0.01 [0.01-0.02] | -1.36 [-1.72 to -1] |
| Iceland | 0 [0-0] | 0 [0-0] | NaN | 0.02 [0.01-0.03] | 0 [0-0.01] | -6.02 [-6.38 to -5.65] |
| India | 6.84 [2.67-13.14] | 11.92 [5.19-20.75] | 74.27 | 0.32 [0.12-0.6] | 0.2 [0.09-0.35] | -1.57 [-1.69 to -1.45] |
| Indonesia | 0.31 [0.13-0.67] | 0.61 [0.28-1.26] | 96.77 | 0.05 [0.02-0.1] | 0.05 [0.02-0.09] | 0.03 [-0.1 to 0.17] |
| Iran (Islamic  Republic of) | 0.01 [0-0.02] | 0.03 [0.01-0.04] | 200.00 | 0.01 [0-0.01] | 0.01 [0-0.01] | 0.46 [-0.24 to 1.16] |
| Iraq | 0.01 [0-0.01] | 0 [0-0.01] | −100.00 | 0.01 [0-0.02] | 0 [0-0] | -5.06 [-5.69 to -4.43] |
| Ireland | 0 [0-0] | 0 [0-0] | NaN | 0.01 [0-0.02] | 0 [0-0] | -14.5 [-16.92 to -12.01] |
| Israel | 0 [0-0] | 0 [0-0] | NaN | 0 [0-0] | 0 [0-0] | -7.06 [-9.85 to -4.19] |
| Italy | 0.05 [0.03-0.06] | 0.05 [0.02-0.06] | 0.00 | 0.01 [0-0.01] | 0 [0-0.01] | -1.25 [-2.31 to -0.19] |
| Jamaica | 0.01 [0-0.01] | 0.02 [0.01-0.03] | 100.00 | 0.07 [0.05-0.1] | 0.11 [0.06-0.16] | 2.09 [1.75 to 2.43] |
| Japan | 0.03 [0.02-0.04] | 0.05 [0.03-0.07] | 66.67 | 0 [0-0] | 0 [0-0] | -1.11 [-2.82 to 0.64] |
| Jordan | 0 [0-0] | 0 [0-0] | NaN | 0 [0-0] | 0 [0-0] | -2.13 [-2.33 to -1.92] |
| Kazakhstan | 0.01 [0-0.02] | 0.01 [0.01-0.02] | 0.00 | 0.01 [0.01-0.02] | 0.01 [0.01-0.02] | 0.86 [0.59 to 1.13] |
| Kenya | 0.02 [0.01-0.04] | 0.04 [0.01-0.09] | 100.00 | 0.04 [0.01-0.08] | 0.03 [0.01-0.06] | -1.31 [-1.58 to -1.04] |
| Kuwait | 0 [0-0] | 0 [0-0] | NaN | 0 [0-0] | 0 [0-0] | 4.14 [2.94 to 5.35] |
| Kyrgyzstan | 0 [0-0.01] | 0 [0-0.01] | NaN | 0.02 [0.01-0.03] | 0.01 [0.01-0.02] | -0.97 [-1.2 to -0.74] |
| Lao People's  Democratic  Republic | 0 [0-0.01] | 0.01 [0-0.01] | Inf | 0.02 [0-0.07] | 0.02 [0.01-0.05] | 0.48 [0.2 to 0.77] |
| Latvia | 0 [0-0] | 0 [0-0] | NaN | 0.01 [0-0.01] | 0.01 [0-0.01] | -1.3 [-1.73 to -0.88] |
|  |  |  |  |  |  |  |
|  |  |  |  |  |  |  |
| Lebanon | 0 [0-0] | 0 [0-0] | NaN | 0 [0-0.01] | 0 [0-0] | -0.04 [-0.34 to 0.27] |
| Lesotho | 0 [0-0] | 0 [0-0] | NaN | 0.03 [0.01-0.08] | 0.02 [0.01-0.07] | -0.9 [-1.33 to -0.47] |
| Liberia | 0 [0-0.01] | 0 [0-0] | NaN | 0.04 [0-0.1] | 0.01 [0-0.04] | -4.34 [-4.58 to -4.11] |
| Libya | 0 [0-0] | 0 [0-0] | NaN | 0 [0-0] | 0 [0-0] | 0.19 [-0.37 to 0.74] |
| Lithuania | 0 [0-0] | 0 [0-0] | NaN | 0.01 [0-0.01] | 0 [0-0.01] | -1.39 [-3.19 to 0.45] |
| Luxembourg | 0 [0-0] | 0 [0-0] | NaN | 0 [0-0] | 0 [0-0] | -1.92 [-2.23 to -1.61] |
| Madagascar | 0.01 [0-0.02] | 0.02 [0-0.03] | 100.00 | 0.04 [0.01-0.08] | 0.02 [0.01-0.05] | -2.15 [-2.22 to -2.07] |
| Malawi | 0.01 [0-0.03] | 0.02 [0-0.04] | 100.00 | 0.05 [0.01-0.11] | 0.03 [0.01-0.07] | -2.07 [-2.16 to -1.98] |
| Malaysia | 0 [0-0.01] | 0.01 [0-0.02] | Inf | 0.01 [0-0.01] | 0.01 [0-0.01] | 0.52 [0.2 to 0.85] |
| Maldives | 0 [0-0] | 0 [0-0] | NaN | 0.08 [0.01-0.24] | 0.07 [0.03-0.12] | -0.12 [-0.34 to 0.11] |
| Mali | 0.01 [0-0.03] | 0.01 [0-0.02] | 0.00 | 0.06 [0-0.15] | 0.02 [0-0.05] | -3.68 [-3.86 to -3.5] |
| Malta | 0 [0-0] | 0 [0-0] | NaN | 0 [0-0] | 0 [0-0] | -3.79 [-4.12 to -3.47] |
| Marshall Islands | 0 [0-0] | 0 [0-0] | NaN | 0.03 [0.01-0.06] | 0.03 [0.01-0.06] | 0.2 [0.05 to 0.34] |
| Mauritania | 0 [0-0.01] | 0 [0-0] | NaN | 0.04 [0-0.09] | 0.01 [0-0.03] | -4.35 [-4.51 to -4.19] |
| Mauritius | 0 [0-0] | 0 [0-0] | NaN | 0.01 [0-0.02] | 0 [0-0.01] | -9.98 [-11.06 to -8.9] |
| Mexico | 0.25 [0.15-0.3] | 0.22 [0.15-0.34] | −12.00 | 0.14 [0.07-0.17] | 0.04 [0.03-0.06] | -4.31 [-4.76 to -3.86] |
| Micronesia  (Federated States  of) | 0 [0-0] | 0 [0-0] | NaN | 0.03 [0.01-0.07] | 0.02 [0-0.05] | -0.81 [-0.85 to -0.76] |
| Mongolia | 0.02 [0-0.04] | 0.02 [0-0.04] | 0.00 | 0.18 [0.02-0.42] | 0.09 [0.02-0.19] | -3.13 [-3.55 to -2.71] |
| Montenegro | 0 [0-0] | 0 [0-0] | NaN | 0.03 [0.01-0.06] | 0.01 [0.01-0.03] | -3.13 [-3.29 to -2.97] |
| Morocco | 0 [0-0] | 0 [0-0] | NaN | 0 [0-0] | 0 [0-0] | 1.78 [1.39 to 2.18] |
|  |  |  |  |  |  |  |
|  |  |  |  |  |  |  |
| Mozambique | 0.01 [0-0.02] | 0.02 [0.01-0.04] | 100.00 | 0.04 [0.01-0.07] | 0.03 [0.01-0.06] | -1.07 [-1.15 to -0.99] |
| Myanmar | 0.03 [0.01-0.09] | 0.07 [0.02-0.13] | 133.33 | 0.02 [0.01-0.06] | 0.02 [0.01-0.05] | 1.09 [0.83 to 1.36] |
| Namibia | 0 [0-0] | 0 [0-0.01] | NaN | 0.03 [0.01-0.07] | 0.02 [0.01-0.07] | -0.59 [-0.82 to -0.35] |
| Nepal | 0.19 [0.06-0.44] | 0.3 [0.12-0.6] | 57.89 | 0.39 [0.11-0.97] | 0.26 [0.1-0.52] | -1.57 [-1.85 to -1.3] |
| Netherlands | 0.06 [0.02-0.08] | 0.04 [0.02-0.05] | −33.33 | 0.05 [0.02-0.07] | 0.02 [0.01-0.02] | -2.79 [-3.25 to -2.32] |
| New Zealand | 0.01 [0-0.01] | 0 [0-0] | −100.00 | 0.03 [0.01-0.04] | 0 [0-0.01] | -7.83 [-9.18 to -6.47] |
| Nicaragua | 0 [0-0.01] | 0 [0-0.01] | NaN | 0.02 [0.01-0.04] | 0.01 [0.01-0.03] | -0.77 [-2.18 to 0.65] |
| Niger | 0.01 [0-0.03] | 0.01 [0-0.03] | 0.00 | 0.06 [0-0.16] | 0.03 [0.01-0.08] | -2.5 [-2.66 to -2.35] |
| Nigeria | 0.11 [0.01-0.23] | 0.08 [0.03-0.17] | −27.27 | 0.04 [0-0.08] | 0.02 [0-0.03] | -3.6 [-3.76 to -3.43] |
| North Macedonia | 0 [0-0] | 0 [0-0] | NaN | 0.01 [0-0.02] | 0.01 [0-0.01] | -2.6 [-2.78 to -2.43] |
| Northern Mariana  Islands | 0 [0-0] | 0 [0-0] | NaN | 0.04 [0.02-0.1] | 0.02 [0.01-0.04] | -2.33 [-2.7 to -1.96] |
| Norway | 0.01 [0.01-0.01] | 0 [0-0.01] | −100.00 | 0.02 [0.01-0.03] | 0.01 [0.01-0.01] | -6.94 [-8.43 to -5.44] |
| Oman | 0 [0-0] | 0 [0-0] | NaN | 0 [0-0] | 0 [0-0] | -3.04 [-3.34 to -2.74] |
| Pakistan | 1.48 [0.38-3.92] | 1.83 [0.8-3.5] | 23.65 | 0.55 [0.13-1.57] | 0.3 [0.12-0.58] | -2.26 [-2.34 to -2.18] |
| Palestine | 0 [0-0] | 0 [0-0] | NaN | 0 [0-0] | 0 [0-0] | -3.11 [-3.89 to -2.32] |
| Panama | 0.01 [0-0.01] | 0 [0-0] | −100.00 | 0.07 [0.01-0.09] | 0 [0-0.01] | -11.12 [-12.94 to -9.25] |
| Papua New Guinea | 0 [0-0] | 0.01 [0-0.02] | Inf | 0.02 [0-0.04] | 0.02 [0-0.04] | 1.07 [0.98 to 1.15] |
| Paraguay | 0 [0-0.01] | 0.01 [0-0.01] | Inf | 0.03 [0.01-0.05] | 0.02 [0-0.03] | -2.73 [-3.98 to -1.46] |
| Peru | 0.07 [0.02-0.11] | 0.06 [0.03-0.16] | −14.29 | 0.11 [0.04-0.17] | 0.03 [0.02-0.09] | -5.03 [-5.55 to -4.5] |
| Philippines | 0.05 [0.02-0.12] | 0.14 [0.05-0.25] | 180.00 | 0.02 [0.01-0.06] | 0.03 [0.01-0.05] | 1.01 [0.72 to 1.29] |
|  |  |  |  |  |  |  |
|  |  |  |  |  |  |  |
|  |  |  |  |  |  |  |
| Poland | 0.05 [0.01-0.05] | 0 [0-0.02] | −100.00 | 0.02 [0-0.03] | 0 [0-0] | -13.63 [-15.4 to -11.81] |
| Portugal | 0 [0-0.01] | 0 [0-0.01] | NaN | 0.01 [0-0.01] | 0 [0-0] | -4.84 [-6.18 to -3.48] |
| Puerto Rico | 0.01 [0-0.01] | 0 [0-0.01] | −100.00 | 0.04 [0.02-0.05] | 0.01 [0.01-0.03] | -4.42 [-5.23 to -3.61] |
| Qatar | 0 [0-0] | 0 [0-0] | NaN | 0 [0-0] | 0 [0-0] | -0.4 [-0.76 to -0.04] |
| Republic of Korea | 0.05 [0.02-0.07] | 0.04 [0.02-0.1] | −20.00 | 0.03 [0.01-0.04] | 0.01 [0.01-0.02] | -4.41 [-4.68 to -4.13] |
| Republic of  Moldova | 0 [0-0] | 0 [0-0] | NaN | 0.01 [0-0.01] | 0 [0-0] | -4.55 [-4.9 to -4.19] |
| Romania | 0.03 [0.01-0.04] | 0 [0-0.01] | −100.00 | 0.02 [0-0.03] | 0 [0-0] | -9.73 [-11.23 to -8.21] |
| Russian Federation | 0.15 [0.09-0.2] | 0.12 [0.08-0.16] | −20.00 | 0.01 [0.01-0.02] | 0.01 [0.01-0.01] | -1.63 [-1.92 to -1.35] |
| Rwanda | 0.01 [0-0.02] | 0.01 [0-0.03] | 0.00 | 0.05 [0.01-0.11] | 0.03 [0.01-0.06] | -1.96 [-2.13 to -1.8] |
| Saint Lucia | 0 [0-0] | 0 [0-0] | NaN | 0.01 [0-0.01] | 0 [0-0] | -4.94 [-5.9 to -3.97] |
| Saint Vincent and  the Grenadines | 0 [0-0] | 0 [0-0] | NaN | 0.02 [0.01-0.05] | 0.03 [0.02-0.04] | -1.16 [-2.17 to -0.15] |
| Samoa | 0 [0-0] | 0 [0-0] | NaN | 0.02 [0.01-0.03] | 0.02 [0-0.04] | 0.36 [0.22 to 0.49] |
| Sao Tome and  Principe | 0 [0-0] | 0 [0-0] | NaN | 0.04 [0.02-0.12] | 0.04 [0.01-0.12] | -0.37 [-0.61 to -0.13] |
| Saudi Arabia | 0 [0-0] | 0 [0-0] | NaN | 0 [0-0.01] | 0 [0-0] | -1.94 [-2.25 to -1.64] |
| Senegal | 0.01 [0-0.02] | 0.01 [0-0.01] | 0.00 | 0.04 [0-0.1] | 0.02 [0-0.04] | -3.49 [-3.66 to -3.32] |
| Serbia | 0.01 [0.01-0.02] | 0.01 [0.01-0.02] | 0.00 | 0.02 [0.01-0.04] | 0.02 [0.01-0.03] | -0.55 [-0.68 to -0.41] |
| Seychelles | 0 [0-0] | 0 [0-0] | NaN | 0.03 [0.02-0.07] | 0.02 [0.01-0.06] | -0.69 [-0.88 to -0.5] |
| Sierra Leone | 0 [0-0.01] | 0 [0-0.01] | NaN | 0.03 [0-0.08] | 0.01 [0-0.04] | -3.57 [-3.73 to -3.4] |
| Singapore | 0 [0-0] | 0 [0-0] | NaN | 0.02 [0.01-0.03] | 0.01 [0-0.01] | -3.69 [-4.21 to -3.17] |
|  |  |  |  |  |  |  |
|  |  |  |  |  |  |  |
| Slovakia | 0.01 [0-0.02] | 0.01 [0-0.01] | 0.00 | 0.04 [0.02-0.07] | 0.01 [0.01-0.04] | -3.64 [-3.98 to -3.3] |
| Slovenia | 0 [0-0] | 0 [0-0] | NaN | 0.02 [0.01-0.02] | 0 [0-0] | -8.08 [-8.77 to -7.37] |
| Solomon Islands | 0 [0-0] | 0 [0-0] | NaN | 0.04 [0.01-0.1] | 0.04 [0.01-0.08] | -0.11 [-0.19 to -0.03] |
| Somalia | 0.01 [0-0.02] | 0.02 [0-0.05] | 100.00 | 0.06 [0.01-0.14] | 0.05 [0.01-0.11] | -0.49 [-0.68 to -0.31] |
| South Africa | 0.05 [0.01-0.08] | 0.09 [0.03-0.13] | 80.00 | 0.04 [0.01-0.07] | 0.04 [0.01-0.06] | -0.61 [-0.88 to -0.34] |
| South Sudan | 0 [0-0.01] | 0.01 [0-0.01] | Inf | 0.04 [0.01-0.08] | 0.03 [0.01-0.06] | -0.92 [-1 to -0.84] |
| Spain | 0.02 [0.01-0.02] | 0.01 [0.01-0.03] | −50.00 | 0.01 [0-0.01] | 0 [0-0] | -4.52 [-5.56 to -3.46] |
| Sri Lanka | 0.03 [0.02-0.07] | 0.03 [0.01-0.1] | 0.00 | 0.05 [0.03-0.11] | 0.03 [0.01-0.08] | -2.3 [-2.64 to -1.96] |
| Sudan | 0 [0-0.01] | 0 [0-0.01] | NaN | 0 [0-0.01] | 0 [0-0] | 0.43 [0.12 to 0.73] |
| Suriname | 0 [0-0] | 0 [0-0] | NaN | 0.02 [0.01-0.04] | 0.02 [0.01-0.03] | -1.3 [-1.58 to -1.02] |
| Sweden | 0.01 [0.01-0.01] | 0.01 [0.01-0.02] | 0.00 | 0.01 [0.01-0.01] | 0.01 [0-0.01] | -1.16 [-2.21 to -0.11] |
| Switzerland | 0 [0-0] | 0.02 [0-0.02] | Inf | 0 [0-0] | 0.01 [0-0.02] | 9.09 [5.75 to 12.54] |
| Syrian Arab  Republic | 0.08 [0.02-0.18] | 0.06 [0.03-0.12] | −25.00 | 0.13 [0.04-0.26] | 0.07 [0.03-0.14] | -1.29 [-1.53 to -1.05] |
| Tajikistan | 0.02 [0.01-0.05] | 0.04 [0.01-0.07] | 100.00 | 0.13 [0.04-0.3] | 0.11 [0.04-0.19] | -0.33 [-0.72 to 0.06] |
| Thailand | 0.05 [0.03-0.1] | 0.05 [0.02-0.11] | 0.00 | 0.02 [0.01-0.04] | 0.01 [0.01-0.02] | -3.95 [-4.49 to -3.41] |
| Timor-Leste | 0 [0-0] | 0 [0-0] | NaN | 0.02 [0-0.07] | 0.03 [0.01-0.07] | 0.83 [0.62 to 1.04] |
| Togo | 0 [0-0.01] | 0 [0-0.01] | NaN | 0.04 [0-0.1] | 0.01 [0-0.03] | -3.95 [-4.14 to -3.76] |
| Tonga | 0 [0-0] | 0 [0-0] | NaN | 0.02 [0.01-0.03] | 0.02 [0-0.04] | 0.53 [0.42 to 0.64] |
| Trinidad and  Tobago | 0 [0-0.01] | 0 [0-0.01] | NaN | 0.1 [0.05-0.14] | 0.04 [0.03-0.07] | -2.66 [-3.17 to -2.15] |
| Tunisia | 0 [0-0] | 0 [0-0] | NaN | 0 [0-0] | 0 [0-0] | 0.81 [0.41 to 1.21] |
|  |  |  |  |  |  |  |
|  |  |  |  |  |  |  |
|  |  |  |  |  |  |  |
| Turkey | 0.06 [0.03-0.11] | 0.08 [0.03-0.14] | 33.33 | 0.03 [0.01-0.06] | 0.02 [0.01-0.03] | -1.23 [-1.74 to -0.73] |
| Turkmenistan | 0 [0-0] | 0 [0-0] | NaN | 0.02 [0.01-0.03] | 0.01 [0.01-0.02] | -1.67 [-1.88 to -1.46] |
| Uganda | 0.02 [0-0.05] | 0.03 [0.01-0.07] | 50.00 | 0.05 [0.01-0.13] | 0.03 [0.01-0.06] | -1.96 [-2.08 to -1.83] |
| Ukraine | 0.02 [0.01-0.04] | 0.02 [0.01-0.03] | 0.00 | 0 [0-0.01] | 0 [0-0.01] | -0.21 [-0.51 to 0.1] |
| United Arab  Emirates | 0 [0-0] | 0 [0-0] | NaN | 0 [0-0] | 0 [0-0] | -0.36 [-0.74 to 0.02] |
| United Kingdom | 0.14 [0.08-0.17] | 0.06 [0.03-0.13] | −57.14 | 0.02 [0.01-0.03] | 0.01 [0-0.02] | -3.71 [-4.03 to -3.39] |
| United Republic of  Tanzania | 0.02 [0.01-0.04] | 0.04 [0.01-0.08] | 100.00 | 0.04 [0.01-0.06] | 0.02 [0.01-0.05] | -1.32 [-1.45 to -1.2] |
| United States of  America | 0.16 [0.09-0.19] | 0.21 [0.14-0.37] | 31.25 | 0.01 [0-0.01] | 0.01 [0-0.01] | -1.23 [-1.43 to -1.02] |
| United States Virgin  Islands | 0 [0-0] | 0 [0-0] | NaN | 0.01 [0-0.02] | 0 [0-0.01] | -2.47 [-2.63 to -2.31] |
| Uruguay | 0 [0-0] | 0 [0-0] | NaN | 0.01 [0.01-0.02] | 0 [0-0.01] | -4.89 [-5.48 to -4.3] |
| Uzbekistan | 0 [0-0] | 0 [0-0] | NaN | 0 [0-0] | 0 [0-0] | -0.13 [-0.53 to 0.27] |
| Vanuatu | 0 [0-0] | 0 [0-0] | NaN | 0.02 [0.01-0.05] | 0.02 [0-0.05] | -0.12 [-0.32 to 0.09] |
| Venezuela  (Bolivarian Republic  of) | 0.02 [0.01-0.03] | 0.01 [0.01-0.03] | −50.00 | 0.03 [0.01-0.06] | 0.01 [0-0.02] | -4.68 [-5.27 to -4.1] |
| Viet Nam | 0.01 [0-0.02] | 0.01 [0.01-0.04] | 0.00 | 0 [0-0.01] | 0 [0-0.01] | -0.71 [-0.93 to -0.49] |
| Yemen | 0 [0-0] | 0 [0-0.01] | NaN | 0 [0-0.01] | 0 [0-0] | 0.57 [0.09 to 1.04] |
| Zambia | 0.01 [0-0.02] | 0.02 [0-0.03] | 100.00 | 0.05 [0.01-0.11] | 0.03 [0.01-0.06] | -2 [-2.05 to -1.95] |
| Zimbabwe | 0 [0-0.02] | 0.01 [0-0.03] | Inf | 0.02 [0.01-0.1] | 0.02 [0-0.08] | 0.25 [-0.15 to 0.65] |
|  |  |  |  |  |  |  |
