# Supplementary material for: Global burden and trends of pelvic organ prolapse associated with aging women: An observational trend study from 1990 to 2019
Source: Front Public Health. 2022 Sep 15;10:975829. doi: 10.3389/fpubh.2022.975829 (PMC9521163; doi:10.3389/fpubh.2022.975829)
Supplement: Supplementary Table 4 — Literature review on risk factors of POP and its recurrence. [file Data_Sheet_4.docx]

Supplementary Table4. Literature review on risk factors of POP and its recurrence

| Title | Sample size | Significant risk factors (P value < 0.05) | Statistical method |
| --- | --- | --- | --- |
| Online prediction tool for female pelvic floor dysfunction: development and validation | 5,545 | Age, Parity, Vaginal delivery | t-test |
| Predictors for pelvic organ prolapse recurrence after sacrocolpopexy: A matched case-control study | 1,102 | Age, POPQ (genital hiatus) | Pearson χ^2^ test, Fisher’s exact test, t-test |
| A predictive model of choosing pessary type for women with symptomatic pelvic organ prolapse | 394 | POP-Q stage, Hiatal area on Valsalva, Hiatal circumference on Valsalva | Mann-Whitney U test, multivariate logistic regression |
| Prevalence, risk factors, and predictors of pelvic organ prolapse: a community-based study | 557 | Age, Parity, Miscarriage, Menopause status, Heigh, Weight, BMI, Cigarettes smoked | t-test, χ^2^ test |
| Prediction of postpartum pelvic floor dysfunction with a nomogram model based on big data collected during pregnancy | 1,500 | Age, History of constipation, History of childbirth, Urinary incontinence during pregnancy, Biparietal diameter at 32 weeks of gestation | Fisher’s exact test, t-test, χ^2^ test, |
| Risk index for pelvic organ prolapse based on established individual risk factors | 906 | Age, Menopause, Parity, Vaginal deliveries, Difficult obstetric history, BMI, Heavy lifting, Family history of prolapse or connective tissue disorders, Varicose veins, Haemorrhoids | Multivariate regression analysis |
| Race as a predictor of urinary incontinence and pelvic organ prolapse | 315 | Age, BMI, Parity, Menopause, Estrogen therapy, Tobacco, Diabetes, Hypertension, Hysterectomy, Continence surgery, Prolapse surgery | χ^2^ test, Fisher’s exact tes, t-test |
| Predictive factors for pelvic organ prolapse (POP) in Iranian women’s: An ordinal logistic approch | 365 | Age, BMI, Maximum birth weight, Pregnancy number, Pelvic surgical number, Occupation (Heavy work), Delivery operative, Delivery position, Delivery Mode | χ^2^ test, t-test,liner regression, logistic regression |
